# The evolution of pelvic limb muscle moment arms in bird-line archosaurs

**DOI:** 10.1126/sciadv.abe2778

**Published:** 2021-03-19

**Authors:** V. R. Allen, B. M. Kilbourne, J. R. Hutchinson

**Affiliations:** 1 Structure and Motion Laboratory, Department of Comparative Biomedical Sciences, The Royal Veterinary College, Hawkshead Lane, North Mymms, Hertfordshire AL9 7TA, UK.; 2 Museum für Naturkunde Berlin, Leibniz Institut für Evolutions-und Biodiversitätsforschung, Invalidenstraße 43, 10115 Berlin, Germany.

## Abstract

Bipedal locomotion evolved along the archosaurian lineage to birds, shifting from “hip-based” to “knee-based” mechanisms. However, the roles of individual muscles in these changes and their evolutionary timings remain obscure. Using 13 three-dimensional musculoskeletal models of the hindlimbs of bird-line archosaurs, we quantify how the moment arms (i.e., leverages) of 35 locomotor muscles evolved. Our results support two hypotheses: From early theropod dinosaurs to birds, knee flexors’ moment arms decreased relative to knee extensors’, and medial long-axis rotator moment arms for the hip increased (trading off with decreased hip abductor moment arms). Our results reveal how, from the Triassic Period, bipedal theropod dinosaurs gradually modified their hindlimb form and function, shifting more from hip-based to knee-based locomotion and hip-abductor to hip-rotator balancing mechanisms inherited by birds. Yet, we also discover unexpected ancestral specializations in larger Jurassic theropods, lost later in the bird-line, complicating the paradigm of gradual transformation.

## Teaser

Biomechanical modeling shows that archosaur leg muscle leverage evolved gradually, yet with a Jurassic pulse of specialization.

## INTRODUCTION

The evolution of terrestrial locomotion in “ruling reptiles” (Archosauria) across the Mesozoic era is an important macroevolutionary event because of the major innovations in form and function during divergence from the original quadrupedal, possibly semisprawling ancestor, most prominent in dinosaurs (*1*, *2*). Fossil skeletons and trackways indicate that the earliest theropod dinosaurs were already striding obligate bipeds with relatively adducted, extended hindlimb postures (*3*, *4*). The presence of a large tail and a robust femoral fourth trochanter in these taxa supports the inference that early dinosaurs used a similar pelvic limb retraction mechanism to living (nonavian) saurians: retracting the entire limb around the hip using large caudofemoral and other muscles [“hip-based” locomotion; (*4*, *5*)]. At some point along the evolutionary lineage leading to crown group birds (the “bird-line” archosaurian reptiles), this was replaced by the mechanism seen in extant birds: retraction of the elongate portion of the limb distal to the knee using the “hamstring” muscles, with the femur habitually held subhorizontal and hip motion greatly de-emphasized [“knee-based” locomotion; (*4*, *6*, *7*)]. Trackway evidence is consistent with an early shift in Triassic theropod dinosaurs to adopting another characteristic of locomotion in living birds: a walk-to-run gait transition that is continuous (involving a “grounded run” at intermediate speeds) rather than discrete (as in humans) (*8*). This evidence reinforces the idea that some key aspects of avian locomotion are very ancient, but the antiquity versus novelty of many specific musculotendinous mechanisms that produced general changes in archosaurian locomotion on the bird-line remains mysterious.

**Fig. 1:**
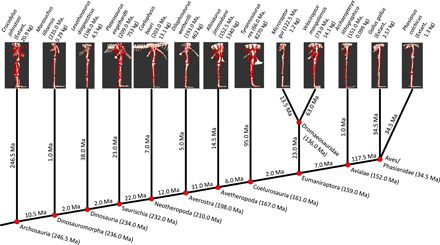
Phylogeny of Archosauria (see text for references), with key nodes connecting the lineages of 13 modeled taxa to the ancestral conditions estimated in this study. Branch lengths (to modeled taxa; and between nodes) and divergence times (at nodes with clade names) are shown. Mean (or measured, for extant taxa) body masses of modeled taxa are in parentheses with branch lengths. Ma, million years.

Analysis of the available anatomical evidence has built up a reasonably detailed account of the gradual transition between hip-based and knee-based locomotion in bird-line dinosaurs (*9*). Tail shortening, anatomical changes in the proximal tail base and pelvis, and reduction of the size of the femoral fourth trochanter indicate that the relative size of the *M. caudofemoralis longus* (CFL, the major caudofemoral muscle) was greatly reduced in maniraptoran theropods (*4*) (i.e., before the origin of flight or birds). Within Jurassic stem birds (early Avialae), further tail shortening, the development of a fused pygostyle, and, lastly in Cretaceous Ornithurae (i.e., close to crown group birds, Aves), the convergence of the iliac blades at the dorsal sagittal midline indicate functional isolation of the tail from the pelvic limb for use as a flight surface (*4*, *10*, *11*). This places the broad phylogenetic domain of de-emphasis of the hip-based limb retraction system as occurring between the nodes Neotheropoda and Aves (Fig. 1).

Evidence for the timing of the adoption of knee-based locomotion is less direct. This inference also depends on the (parsimonious) assumption of a continuous spectrum between hip- and knee-based locomotion without apomorphic intermediate states, which is yet to be tested via quantitative biomechanical analysis. Between the nodes Neotheropoda and Aves, the estimated position of the whole-body center of mass (CoM) shifted cranially, correlated with tail reduction and the evolution of large pectoral limbs (both of which effectively shifted mass toward the front of the animal), with much of this shift thought to have occurred within Coelurosauria (*4*, *12*). Toward the middle of the support phase (“midstance”), the ground-reaction force (GRF) vector of a steady-state striding bipedal tetrapod is more or less vertically oriented between the center of pressure in the foot and the CoM (*13*). The position of the CoM constrains femoral posture (at least around midstance), as the knee joint tends to be placed cranial to the CoM in order for the knee extensor muscles to provide support forces to counter the GRF [(*14*, *15*), but see (*16*)].

Craniad shifts in whole-body CoM are therefore evidence of gradual adoption of a derived, subhorizontally inclined femur between Neotheropoda and Aves (*12*). Additional evidence comes from the iliac antitrochanter, which shifted from an ancestral position dorsal to the acetabulum to one caudodorsal to it between Tetanurae and extant birds (*2*, *9*, *17*). The antitrochanter articulated with the proximal-most surface of the femoral neck, and so a more caudodorsal position indicates that the femur itself was oriented more subhorizontally. Furthermore, studies of theropod limb proportions reveal that metatarsal (sole) bones became relatively longer between Maniraptora and Avialae (*18*–*20*). In knee-based locomotion, the femur moves less, and so contributes less to stride length (effectively shortening the limb). Elongation of the distal limb segments could compensate for this reduction.

Last, possibly the best clues for locomotor evolution on the bird-line are found in the evolution of muscular mechanisms used for balance. In steady-state locomotion, the GRF associated with the body’s weight and inertia acts (on average) within the sagittal midline plane. As bipeds such as avian and nonavian theropods are monopedal when striding quickly (i.e., aerial running), to maintain balance, they must place their feet at (or close to) the sagittal midline, medial to the hip. This medial offset grants the GRF a moment arm to create frontal plane moments about the hip that would collapse the limb by overadduction if not balanced by abduction moments of comparable magnitude (*2*). A shift in habitual femoral orientation during the support phase from subvertical (in hip-based locomotion) to subhorizontal (in knee-based locomotion) aligns the femoral long axis close to perpendicular to the frontal plane. This alters how the frontal-plane moment affects the hip joint, such that the whole-limb adduction force manifests as a lateral (external) moment about the femoral long axis. Extant birds therefore balance more by controlling rotation about the femoral long axis [particularly by applying medial (internal) rotation moments to prevent limb collapse] than by controlling adduction/abduction of the hip [much of which is passively constrained by the iliac antitrochanter; (*2*, *9*, *17*)]. Hence, the femora of birds are loaded heavily in torsional twisting rather than bending (*19*).

Qualitative analysis of bird-line hip anatomy suggests that enhanced control of hip long-axis rotation (LAR) evolved between Neotheropoda and Eumaniraptora (close to Avialae), with the implication that ancestral eumaniraptorans were capable of maintaining balance with a more horizontally inclined femur (*2*). Subsequent anatomical changes are more subtle but indicate that the anatomy underpinning limb-balancing mechanisms was virtually identical to that of crown group birds by at least Ornithurae (*2*, *12*, *21*).

The available evidence supports the hypothesis that knee-based locomotion gradually replaced hip-based locomotion along the bird-line between Neotheropoda and Ornithurae/Aves (*9*, *22*). Furthermore, the coincidence of appreciable transformations in tail-base anatomy, femoral long-axis control mechanisms, whole-body CoM, and limb proportions in Neotheropoda suggests that trends in terrestrial locomotor evolution were accelerated within this clade.

However, as stated, much of the inferences from muscle function rest on qualitative assessments of muscle moment arms (leverage) based on simple observations. Muscle sizes are also important, and their evolution has been qualitatively gauged from bone dimensions [e.g., (*5*, *16*, *23*)] or even quantitatively estimated (*12*). All other factors being equal, larger moment arms tend to increase joint moments but decrease joint rotational velocity or mobility. Computational methods for assessing muscle moment arms (and other aspects of muscle function) are well established in biomechanics research (*24*, *25*) and have been used to analyze limb muscle function in extinct dinosaurs (*26*–*29*), but not yet in an explicit, quantitative phylogenetic context. The study of the evolution of bipedalism in theropod dinosaurs and their relatives within Archosauria would benefit from such quantitative analysis.

**Fig. 2:**
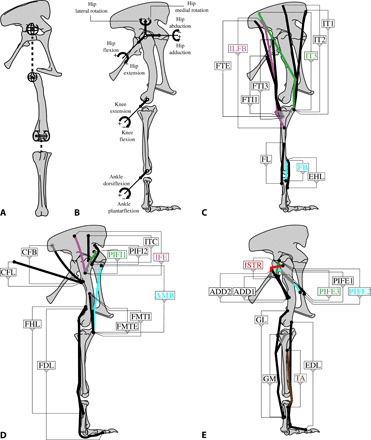
Musculoskeletal model setup [following (*25*, *26*); also see (*16*)]. *Allosaurus* right hindlimb in side view for example. (**A**) Examples of fitting geometric primitives (spheres for hip; cylinders for knee; also ankle) to position joints and determine joint centers of rotation. (**B**) Coordinate system adopted (in reference pose with all joint angles = 0°), showing main degrees of freedom allowed in models and positive/negative signs of angles and moment arms. (**C**) Superficial and (**D** and **E**) deeper layers of muscle-tendon unit (MTU) paths (origins to insertions); details for acronyms are in Table 1. Open circles/gray lines depict hidden (more medial) attachments/lines of action.

With the above potential in mind, here, we estimated moment arms for all (35) major pelvic limb muscles from computational musculoskeletal models (Fig. 2; Table 1) of 13 key archosaurian taxa, particularly on the bird-line [including two extant birds; Phasianidae (Galliformes)]. We used these moment arms to investigate the prevailing idea that bird-line archosaurs switched from hip-based to knee-based locomotion between Archosauria (especially Neotheropoda) and Aves. To guide our analysis, we developed the following hypotheses regarding pelvic limb moment arms.

### Hypothesis 1

In knee-based locomotion, the hip moves little during stance phase [e.g., ~5° to 10° in walking guineafowl, versus 60° to 80° in walking juvenile alligators; (*4*, *6*)], but large hip extension moments must still be exerted for weight support, because the CoM is more cranially located from the hip, resulting from a trend between the Neotheropoda and Aves nodes (*12*). Large moment arms for hip extension would be advantageous. Hip extension moment arms therefore should generally have increased between Neotheropoda and Aves. However, craniad expansion of the preacetabular ilium should also have increased hip flexion moment arms (*5*, *26*–*28*), and so we also predict that the ratio of moment arms for hip extension to those for hip flexion remained constant along the archosaurian bird-line.

### Hypothesis 2

In knee-based locomotion, the knee flexes and extends more [e.g., ~45° to 65° stance phase flexion in walking guineafowl; (*6*)] than in hip-based locomotion [e.g., ~30° to 40° stance phase extension in juvenile alligators; (*4*)]. All else being equal, smaller moment arms for knee flexion would be advantageous for actuating such large arcs (*25*, *29*). Knee flexion moment arms for the hamstring muscles therefore should generally have decreased between Neotheropoda and Aves. As a hypothesized benefit of the subhorizontal femur in knee-based locomotion is to keep the knee close to the CoM (and so maintain a similar flexion moment arm for the GRF), we see no basis for predicting alteration of knee extension moment arms with the adoption of knee-based locomotion. We therefore predict that the ratio of moment arms for knee flexion to those for knee extension decreased between Neotheropoda and Aves.

### Hypothesis 3

Control of frontal plane balance involves exerting large moments to prevent undesirable joint rotations and mediolateral limb instability (*2*). Larger moment arms would be advantageous for generating these moments. Considering that control of balance changed in the shift to knee-based locomotion, moment arms for hip LAR (particularly those for medial rotation to oppose limb collapse) should therefore have generally increased between Neotheropoda and Aves. As muscular control of hip abduction is less important in knee-based locomotion, we therefore also expect the ratio of moment arms for hip medial LAR to those for hip abduction to have increased over the same phylogenetic range. An analogous pattern was inferred for muscle moments by Bishop *et al.* (*22*), albeit at a coarser phylogenetic resolution.

In addition to reconstructing changes in moment arm ratios, we also provide a comprehensive synthesis of the evolution of pelvic limb muscle leverages over the bird-line. This overview addresses other ideas at least implicit in the literature on the evolution of archosaurian locomotion along the line to birds. By doing so, our study transcends a focus simply on the transition from hip- to knee-based locomotion, which is vaguely understood to have been gradual (*2*, *4*, *5*, *9*) to reveal how (biomechanically and quantitatively, in a morphofunctional context) and when (phylogenetically) 35 individual muscles (including but not limited to CFL) contributed to that general transition—and how well a gradual evolutionary paradigm fits our data.

## RESULTS

**Fig. 3:**
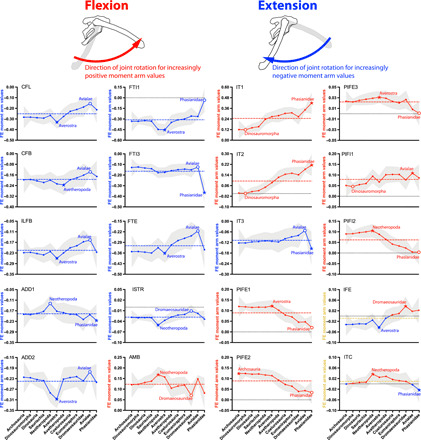
Mean MTU moment arms (normalized by femur length) for hip flexion (red, positive values) and extension (blue, negative values) for the 20 muscles (Fig. 2, C to E, and Table 1) acting around the hip in all taxa, plotted across the ancestral nodes in Fig. 1. Dashed horizontal line represents the average moment arm across all nodes; colored gold if moment arm switches sign. Stars indicate peak moment arm (in either negative or positive orientation; both if sign switches); open circle indicates minimal value (closest to zero) for muscles that do not switch sign. Note that Dromaeosauridae is not on the main line Archosauria-to-Aves/Phasianidae but is a side lineage uniting two deinonychosaurs (Fig. 1); kept for completeness.

**Fig. 4:**
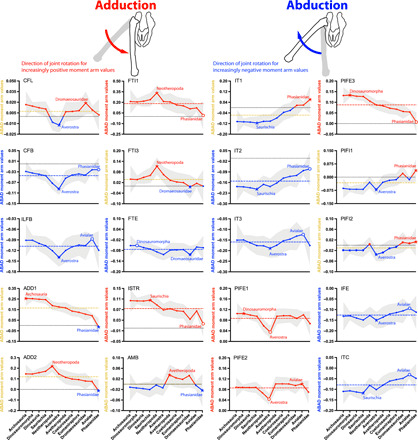
Mean MTU moment arms (normalized by femur length) for hip adduction (red, positive values) and abduction (blue, negative values) for the 20 muscles (Fig. 2, C to E, and Table 1) acting around the hip in all taxa, plotted across the ancestral nodes in Fig. 1. See Fig. 3 caption for further details.

**Fig. 5:**
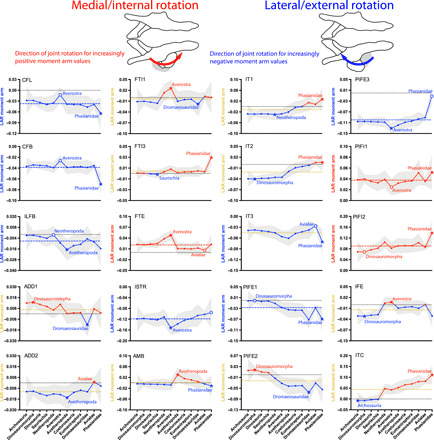
Mean MTU moment arms (normalized by femur length) for long-axis rotation (LAR) of the hip in medial/internal (red, positive values) and lateral/external (blue, negative values) directions for the 20 muscles (Fig. 2, C to E, and Table 1) acting around the hip in all taxa, plotted across the ancestral nodes in Fig. 1. See Fig. 3 caption for further details.

**Fig. 6:**
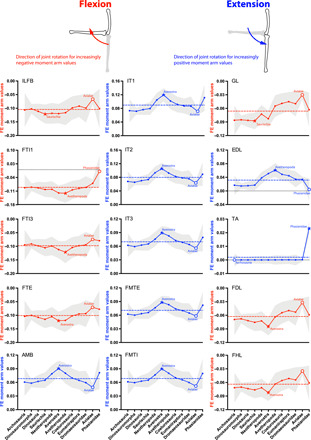
Mean MTU moment arms (normalized by tibiotarsus length) for knee flexion (red, negative values) and extension (blue, positive values) for the 15 muscles (Fig. 2, C to E, and Table 1) acting around the knee in all taxa (if present), plotted across the ancestral nodes in Fig. 1. See Fig. 3 caption for further details.

**Fig. 7:**
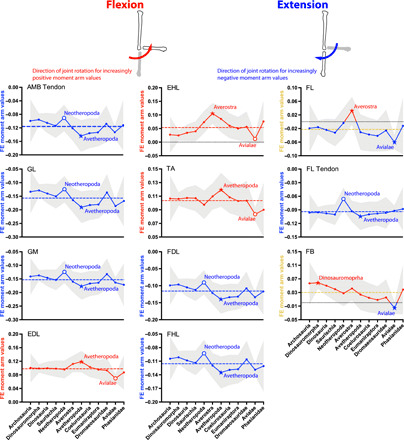
Mean MTU moment arms (normalized by tarsometatarsus length) for ankle flexion (red, positive values) and extension (blue, negative values) for the 11 muscles (Fig. 2, C to E, and Table 1) acting around the ankle in all taxa (if present), plotted across the ancestral nodes in Fig. 1. See Fig. 3 caption for further details.

Unless otherwise stated, all quoted values are individual musculo-tendon unit (MTU) moment arms normalized by segment length, for ancestral nodes along the phylogeny (Fig. 1), given as mean ancestral character state estimates (ACEs) (as plotted in Figs. 3 to 7) across the phylogeny from *fastAnc* reconstructions (see Materials and Methods). Peak and minimal values are provided in text S1 for additional reference. Ninety-five percent confidence intervals are shown in Figs. 3 to 7 (shaded areas), and their quantitative values are in the Supplementary Materials (“data_processing_files” folder: “[hip/knee/ankle]_[Ex/Ab/Ro]_ACE_data_from_dino_moment_arms_Sun_Jun_07_14–26-54_2020-MAC_2.csv”; total of five files). Here, we present the main trends indicated by the ACEs, but if pertinent, note where the 95% confidence intervals render these more ambiguous.

### Hip extensors (Fig. 3)

*M. caudofemoralis longus* and *brevis* (CFL and CFB, means −0.25 and −0.19) showed a similar overall reduction of extensor moment arms over the entire bird-line. Evolutionary trends for *M. iliofibularis* (ILFB, −0.21) were complex but similar to CFL and CFB: Values reached a peak (i.e., most negative) in Averostra/Avetheropoda and then a minimum in Avialae before a slight increase back to Phasianidae. *M. adductor femoris 1* and *2* (ADD1 and ADD2, means −0.19 and −0.24) did not change markedly overall from Archosauria to Phasianidae (a slight increase at most). However, again, there was a peak of values in larger early theropods, with the minimum for ADD1 occurring in Averostra (peak in Phasianidae) whereas the ADD2’s strong peak was in Avetheropoda (minimum in Avialae), an interesting opposing trend.

Trends for the flexor cruris group, *M. flexor tibialis internus 1* (FTI1, mean −0.31), *M. flexor tibialis internus 3* (FTI3, −0.16), and *M. flexor tibialis externus* (FTE, −0.31), varied. FTI1 showed a pattern roughly similar to ADD2’s (sensible given their similar origins) until its loss by the Phasianidae node. FTI3 showed essentially no change and then a sharp increase to a peak value in Phasianidae. FTE and the caudal head of the *M. iliotibialis* group (IT3, −0.11) showed broadly comparable trends to the CFB (above). *M. ischiotrochantericus* (ISTR, −0.028) changed little over the bird-line, remaining a weak hip extensor.

*M. iliofemoralis externus* (IFE, −0.027) switched from a weak hip extensor to a flexor in Coelurosauria. Its counterpart *M. iliotrochantericus caudalis* (ITC, mean −0.0089) varied around zero hip extension capacity, with a mean extensor action in Archosauria, and then becoming a weak flexor for most of dinosauromorph evolution before switching to a weak extensor again in Avialae.

### Hip flexors (Fig. 3)

Hip flexor moment arms were generally smaller (in absolute values) than hip extensors. Estimates of hip flexor moment arms for the *M. ambiens* (AMB, mean 0.12) varied widely across the bird-line, with a minimum in Dromaeosauridae and a peak in Neotheropoda. The two cranial heads of the *M. iliotibialis* group (IT1 and IT2, 0.25 and 0.095) showed steady, strong increases from minima in Dinosauromorpha to peak values in Phasianidae.

The three heads of *M. puboischiofemoralis externus* (PIFE1, PIFE2, and PIFE3; means 0.089, 0.089, and 0.022, respectively) exhibited consistent overall decreases from peak flexor moment arm values in Avetheropoda, Archosauria, and Averostra to minima in Phasianidae (zero value for PIFE3, lost). Last, moment arms for the *M. puboischiofemoralis internus* (PIFI1 and PIFI2, 0.079 and 0.062) differed—the PIFI1 shared the variable trend of AMB, with a minimum in Dinosauromorpha and a peak in Avialae, whereas the PIFI2 slowly increased to a peak in Neotheropoda and then more steeply declined to a very small (or zero) flexor value in Avialae and Phasianidae (related to its line of action very close to the hip, especially in flexed poses).

### Hip adductors (Fig. 4)

Hip adduction moment arms for the CFL (mean 0.011) had patterns grossly similar to its extensor trend (e.g., peak at Averostra) but deviated in that it varied close to a zero adduction moment arm, switching from an adductor in Archosauria to an abductor in Neotheropoda, then back to an adductor in Avetheropoda. Leverages of ADD1 and ADD2 (0.18 and 0.13) overall declined across the bird-line, with ADD2 exhibiting a pronounced peak at Neotheropoda before continuing the decline, and then both ADD1 and ADD2 becoming very weak (or zero-value) hip abductors in Phasianidae. FTI1 and FTI3 (0.18 and 0.046) mimicked the pattern of ADD2, although FTI1 had no moment arm in Phasianidae due to its loss. The ISTR’s adductor moment arm (0.074) decreased over the bird-line, from a peak in Saurischia to a minimum in Phasianidae. The pubic heads PIFE1 and PIFE2 (means 0.087 and 0.086) exhibited only a small net decrease across the bird-line but interestingly declined steeply to minimal values in Averostra. The ischial head PIFE3 (0.088) differed—it had a stronger general decrease from peak values in Dinosauromorpha to zero (loss) in Phasianidae.

### Hip abductors (Fig. 4)

Unlike the CFL, the CFB (mean −0.044) was consistently a weak hip abductor (peak at Averostra; minimum in Phasianidae), but otherwise, its evolutionary pattern was similar to its extensor moment arm’s. This abductor action is opposite that found by Bates *et al.* (*28*). The ILFB (−0.11) had little pronounced change: a peak in Averostra and a minimum in Avialae. Similarly, the FTE (−0.074) exhibited no distinct trend: minimal in Dinosauromorpha versus peaking in Dromaeosauridae.

The AMB (−0.013) showed a peculiar pattern in which it maintained a small adductor moment arm until switching to abduction in Avetheropoda and then back again to adduction in Avialae to Phasianidae. The IT1 and IT2 (−0.086 and − 0.15) maintained steadily decreasing hip abduction moment arms to Phasianidae, and the IT1 even switched into a hip adductor in Dromaeosauridae/Avialae. Contrastingly, the IT3 (−0.16) changed modestly at best, increasing to a peak at Averostra and then decreasing to a minimum at Avialae (reversed somewhat in Phasianidae).

The PIFI1 and PIFI2 (−0.045 and −0.019), although having near-zero moment arms overall, both displayed decreases (after a peak at Averostra, which was a reversal from minimal moment arms at the immediately previous node Neotheropoda), swapping from hip abduction to adduction (peak at Phasianidae), yet the PIFI2’s changes were smaller. The IFE (−0.13) showed even less of a trend, remaining relatively constant at midrange values over the bird-line, with a minor peak at Averostra and a minimum at Avialae. The hip abduction evolutionary trend for the ITC (mean −0.079) was similar to that of the IFE but with a stronger, clear decrease.

### Hip lateral rotators (Fig. 5)

Almost all MTUs (17 of 20) that cross the hip had appreciable moment arms for lateral hip rotation at least in some point of their evolution, with 7 of 20 muscles (IT3, CFB, CFL, ISTR, ILFB, PIFE1, and PIFE3) never clearly switching into medial rotators (see further below). We did not find lateral rotation as a clear action of IT3, unlike (*28*). Hip lateral rotation leverage for the CFL and CFB (means −0.037 and −0.039) presented almost no trends, remaining relatively constant except for slight localized minima in Averostra and peaking in Phasianidae. The ILFB (−0.0072) showed no prominent trend, having only slight lateral rotation capacity. Values for the ADD2 (mean −0.011) decreased marginally over the bird-line (with an isolated switch to medial rotation in Avialae; reversed in Phasianidae).

Moment arms for the FTI1 (mean −0.011) broadly varied close to zero values, remaining relatively constant at small values before decreasing suddenly into medial rotator values in Neotheropoda and Averostra and then reversing back to lateral rotation until reversing again in Avialae. In contrast, the ISTR (−0.12) was a strong lateral rotator, peaking in Averostra and then decreasing slightly to Phasianidae. The AMB (−0.0060) maintained a near-zero, weak moment arm until Avetheropoda, switching sharply into a medial rotator and then gradually decreasing back into a lateral rotator by Avialae. Hip lateral rotation moment arms for the IT1 and IT2 (means −0.021 and −0.026) declined over the bird-line, switching into medial rotation in Dromaeosauridae/Avialae. Values for the caudal head IT3 (mean −0.045) remained relatively constant (with IT3 having a local peak at Avetheropoda) except for a pronounced increase in Phasianidae.

Lateral rotation values for the PIFE1 and PIFE2 (means −0.027 and −0.040) generally started at small values (medial rotation for PIFE2) on the bird-line, but increased across the Neotheropoda to Phasianidae nodes. This prevalence of lateral rotation contrasts with medial rotation for these muscles found by Bates *et al.* (*28*). Moment arms for the PIFE3 (mean −0.10), like the ISTR, generally were much larger, remaining at close to peak values for most of the bird-line before slowly declining beyond the peak at Averostra and then lost in Phasianidae. The IFE (−0.020) decreased its values, even switching into near-zero medial rotation at Neotheropoda and Averostra before reversing that trend (repeated in Avialae; reversed again in Phasianidae).

### Hip medial rotators (Fig. 5)

As noted above, many muscles were medial rotators at certain nodes but lateral rotators in others. The ADD1 (mean 0.0061) remained a very weak medial rotator except for an isolated switch to lateral rotation in Dromaeosauridae. Values for the FTI3 (mean 0.0057) strongly remained close to zero except for a strong peak (with transformation of the FTI3 into the FCM of Aves) in Phasianidae. The FTE’s pattern (0.021) matched the FTI1’s (peaking around Neotheropoda and Averostra), except it was shifted to remain as a weak medial rotator.

However, the PIFI2 (mean 0.090) was consistently a strong medial rotator in our analysis, more so than the PIFI1 (0.038). Values for the PIFI1 and PIFI2 remained mostly steady; PIFI1 did not change appreciably, whereas PIFI2 had a slight overall increasing trend with an increase at Phasianidae. Medial rotation moment arms for the ITC (mean 0.069) showed strong increases overall, in the early bird-line remaining consistently low (indeed, as near-zero lateral rotation) but increasing steadily from Neotheropoda onward, reaching peak values in Phasianidae that rivaled those of the PIFI2 in magnitude.

### Knee extensors (Fig. 6)

Moment arm estimates indicate that the major bird-line knee extensors were as follows: *M. ambiens* (AMB, mean 0.068), the three heads of the *M. iliotibialis* group (IT1, IT2, and IT3, 0.090, 0.080, and 0.070), the two main heads of *M. femorotibialis* (FMTI and FMTE, 0.069 and 0.071), and, lastly, *M. extensor digitorum longus* (EDL, mean 0.032; or else *M. tibialis anterior*, TA in Phasianidae, 0.0020). Note that, unlike at the hip, actions of all of these muscles except the EDL/TA were insensitive to anatomical and kinematic changes at the knee joint, consistently remaining knee extensors across the phylogeny.

Knee extensor moment arms for all MTUs except the EDL showed only weak trends over the bird-line, and even the trend for the EDL was only moderately positive (and reversed). The IT1 to IT3, AMB, FMTE, and FMTI all had distinctly consistent peaks at Averostra and minima at Avialae, with moment arms slightly increasing from Saurischia to the peak and then reversing. The EDL had a roughly similar pattern but with a peak at Avetheropoda and a minimum at Phasianidae (zero moment arm/loss; corresponding to EDL’s loss of a femoral origin and TA’s gain of a femoral origin). These trends were mainly attributable to the anatomically grounded wrapping surfaces used around the knee. For example, the wrap object for IT3, FMTI, FMTE, and AMB had variation (for all 13 models, mean wrap object radius/femur length = 9.47 ± 3.01 SD) relating to the evolutionary anatomy of the distal condyles of the femur but retained consistent proportions (least squares regression through *y* origin: wrap object radius = 0.1421 * femur length; *R*^2^ = 0.945). The peaks for Averostra/Avetheropoda corresponded to (femur length/wrap radius) ratio ~8, versus 17 for *Archaeopteryx*; hence, larger wrapping radii relative to femur length in early (larger) theropods incurred relatively larger moment arms.

### Knee flexors (Fig. 6)

Moment arm estimates indicate that the major bird-line knee flexors were as follows: *M. iliofibularis* (ILFB, mean −0.10), the flexor cruris group including *M. flexor tibialis internus 1* (FTI1, −0.091), *M. flexor tibialis internus 3* (FTI3, −0.098) and *M. flexor tibialis externus* (FTE, −0.10), *M. gastrocnemius lateralis* (GL, −0.066), *M. flexor digitorum longus* (FDL − 0.063), and, lastly, *M. flexor hallucis longus* (FHL, mean −0.064).

Evolutionary trends in knee flexor moment arms followed a very similar pattern for all MTUs. Little change happened for knee flexor moment arms except that their magnitude decreased from peak values around Averostra to a minimum at Avialae, which then reversed toward Phasianidae except for FTI1 (loss in Phasianidae; zero value). The GL differed somewhat in that its moment arm decreased more clearly from Averostra; the FDL and FHL had weaker forms of this trend.

### Ankle plantarflexors (extensors) (Fig. 7)

Moment arm estimates indicate that the major bird-line ankle plantarflexors were as follows: *M. ambiens* (AMB distal tendon, mean −0.12), *M. gastrocnemius lateralis* (GL, −0.16) and *medialis* (GM, −0.15), *M. flexor digitorum longus* (FDL, − 0.12) and *M. flexor hallucis longus* (FHL, −0.12), and, lastly, *M. fibularis longus* (FL, −0.028; with a secondary tendon “FL Tendon”, mean −0.094).

Evolutionary trends in ankle plantarflexor moment arms again followed a similar pattern for most MTUs (AMB, GL, GM, FDL, FL tendon, and FHL): a roughly constant moment arm ACE value with a slight minimum at Neotheropoda and then an increase to Avetheropoda and constancy through to Avialae with possibly a reversal toward Phasianidae. The FL showed an anomalous reversal in Neotheropoda, switching to a small plantarflexor moment arm in Averostra before returning to values more typical of neotheropods, yet overall, its moment arms were small.

### Ankle dorsiflexors (Fig. 7)

Moment arm estimates indicate that the major bird-line ankle dorsiflexors were as follows: *M. extensor digitorum longus* (EDL, mean 0.098), *M. extensor hallucis longus* (EHL, 0.054), *M. tibialis anterior* (TA, 0.10), and, lastly, *M. fibularis brevis* (FB, mean 0.034). The FB mainly decreased its moment arm, switching into a plantarflexor at Avialae but reversing in Phasianidae. The TA and EDL both maintained quasi-constant moment arms, but the EHL’s value varied widely, increasing to Avetheropoda and then declining again to a minimum at Avialae; reversed at Phasianidae.

### Ratios of moment arms (Fig. 8)

**Fig. 8:**
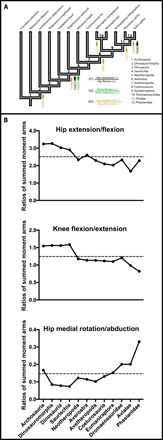
Results for trends in the ratios of summed (absolute value) moment arm ACE values across the phylogeny of Archosauria (see Fig. 1). (A) Changes between nodes (numbers 1 to 12) of ≥20% magnitude are emphasized. Hypothesis 1 (H1) is rejected at Neotheropoda = node 5 (and increase at node 12), H2 is supported at node 5, and H3 is supported at node 5 (and nodes 8, 11, and 12; with unexpected decreases and increases at nodes 2 and 10, respectively). “+” and “–“ signs by muscle actions for H1 to H3 indicate signs of (nonabsolute value) moment arms. LAR, long-axis rotation. (B to D) Detailed changes of ratios of summed moment arms across the phylogeny of Archosauria, used for inferences in (A).

Our Hypotheses 1 to 3 predicted how ratios of summed (absolute value) moment arm ACE values should have evolved on the bird-line (Neotheropoda to Aves/Phasianidae), addressed here. Internodal changes ≥20% are emphasized but similar changes ≥10% are noted here where relevant (see Materials and Methods).

The ratio of summed hip extensor moment arms to summed hip flexor moment arms (mean 2.50) declined 30% over the bird-line. Notable changes occurred as a decrease at the Neotheropoda node, reversed at the Phasianidae node (Fig. 8), but less substantial changes evolved at other nodes, reflecting the general trend of a decrease, as follows (Fig. 3; see data file S3). Hip extensor moment arms overall also increased the ratio +11% in Averostra but then decreased equally in Avetheropoda. Hip flexors overall likewise aided a contrasting increase (+15% in the side-lineage Dromaeosauridae) and decrease (−17% in Avialae) of the ratio. In these cases of increased/decreased extensor or flexor leverage, their antagonists did not exhibit proportional and concurrent changes (see data file S3). Thus, Hypothesis 1, proposing a constant ratio of moment arms, was rejected.

The ratio of summed knee flexor moment arms to summed knee extensor moment arms (mean 1.24) declined 47% over the bird-line. Again, Neotheropoda had the sole major decrease (Fig. 8), but other nodes had appreciable contributions, including decreased moment arms for knee flexion and extension in Avialae (−10% to ratio) and increases of both in Phasianidae (−17% to ratio) (Fig. 7 and data file S4). Hence, Hypothesis 2, that knee flexor/extensor moment arm ratios would decrease, was supported.

The ratio of summed hip medial rotation moment arms to summed hip abduction moment arms (mean 0.15) increased ~100% over the bird-line. This occurred in a notable number of relative “pulses” of change, with an unexpected decrease of the ratio at Dinosauromorpha (caused by enhanced hip abductors versus reduced medial rotators; Figs. 4 and 5 and data file S5), and then a large increase at Neotheropoda followed by smaller ones at Coelurosauria and Avialae (also the side-lineage Dromaeosauridae) and an extra increase at Phasianidae (Fig. 8). There was a smaller change at Eumaniraptora where decreased hip abductor moment arms helped incur a +17% ratio boost (also +11% at Avetheropoda; data file S5). Therefore, Hypothesis 3, a phylogenetic increase of the hip’s medial rotation/abductor ratio, was well supported.

Our sensitivity analysis of the phylogeny’s branch lengths (see Materials and Methods) gave broadly similar results, upholding Hypotheses 2 and 3 but contradicting Hypothesis 1 (fig. S2A). No changes of 20% between nodes were found for the hip extension/flexion ratio (Hypothesis 1). The knee flexion/extension ratio decreased 20% at the Neotheropoda node, much as before (Hypothesis 2). There was a sequence of increases in the hip medial LAR/abduction ratio at the nodes Saurischia, Neotheropoda, Coelurosauria, Eumaniraptora, Avialae, and Phasianidae (as per Hypothesis 3), but the unusual decrease of that ratio at Dinosauromorpha was absent. The shapes of the curves of these summed moment arm ratios also remained similar with the altered branch lengths (fig. S2B), albeit with essentially no decreasing trend for hip extension/flexion (versus Fig. 8B).

While we did not hypothesize major changes in some other moment arms (or their ratios), we found that, from Archosauria to Phasianidae, hip lateral rotators increased 186%, but adductors decreased by almost 75% (Figs. 4 and 5 and data file S5). Thus, changes in antagonists to hip medial rotation/abduction (i.e., Hypothesis 3) mirrored each other. Ankle plantarflexor (extensor) moment arms increased marginally at best (14%), whereas (dorsi)flexors did not alter appreciably overall (Fig. 7and data file S6).

## DISCUSSION

As per Introduction, multiple lines of evidence indicate that the knee-based locomotion of modern birds evolved gradually across the archosaurian bird-line, with transformations concentrated in theropod dinosaurs (*4*, *5*, *9*). While prior studies suggested general conservatism of archosaurian hip moment arms [e.g., (*28*)], out of 20 of our results for each hip degree of freedom (DoF) (Figs. 3 to 5), we found about 2, 6, and 9 evolutionary switches of muscle actions for hip flexion/extension, abduction/adduction, and medial/lateral LAR, revealing some (~28% on average) stark nonconservatism as well. We proposed three hypotheses that should be evident in moment arms of 35 reconstructed pelvic limb muscles along the bird-line, and two of these hypotheses were supported by our results (Fig. 8). The evolution of hip extension/flexion leverage did not fit our Hypothesis 1 (revealing instead remarkable complexity including in Triassic-Jurassic ancestral Neotheropoda and Averostra/Avetheropoda). Yet, we did find that knee flexor leverage reduced whereas knee extensor leverage increased in Neotheropoda, so their ratio supported Hypothesis 2. Furthermore, we uncovered robust support for Hypothesis 3 (regarding ratios of hip medial LAR/abduction), with an exciting result for an initial decrease in bipedal Dinosauromorpha followed by successive increases from Neotheropoda onward on the bird-line. Together, these results add previously unidentified insight into the timing of transformation from hip- to knee-based locomotion and illuminate that changes were concentrated more at some ancestral nodes than others, including specializations in large-bodied Jurassic Averostra/Avetheropoda (and smaller Triassic Neotheropoda) that were lost later in the bird-line. Here, we explore the implications for each hypothesis and then synthesize what they mean for the evolution of terrestrial locomotion across the bird-line.

### Transformations of hip extension/flexion (Hypothesis 1)

The ratio of hip extensor to hip flexor moment arms declined at Neotheropoda and increased at Phasianidae, rejecting Hypothesis 1 (Fig. 8). The evolutionary change was mainly driven by increased (absolute value) hip flexor leverage: +28% in Neotheropoda and +36% overall from Archosauria to Phasianidae (data file S3). As we elaborate on below, the evolution of hip extensor/flexor muscle leverage along the bird-line was far more complex than our Hypothesis 1 anticipated. Our alternative, extreme model of branch lengths also rejected this simple hypothesis (fig. S2).

The estimated early decline in hip extensor/flexor ratios was due to two major trends at the level of individual muscles: First, increases in the moment arms of the hip flexors such as IT1 and IT2 (from Dinosauria onward; Fig. 3), supplemented by the progressively weaker PIFI1 and PIFI2, reflecting cranial expansion of the preacetabular iliac blade from which they originated (*5*). This increase occurred despite decreases to hip flexor moment arms (and likely moment-generating capacities) of synergistic swing-phase muscles such as PIFE1 to PIFE3, attributable to the evolution of pubic/ischial retroversion and “apron” reduction in Coelurosauria/Eumaniraptora (*27*). Second, gradual decreases in the hip extensor moment arms of CFL and CFB across the bird-line (Fig. 3) reflect proximal migration of the femoral fourth trochanter onto which they inserted (*4*). Both increased IT1 and IT2 hip flexor leverage (nodes Dinosauria onward; especially early theropods) and reduction in CFL and CFB hip extensor leverage (past nodes Averostra and Avetheropoda) therefore predated reduction in the relative size of the caudofemoral muscles [from Coelurosauria onward, as indicated by tail anatomy; (*4*, *11*, *12*)] by several phylogenetic nodes. Large muscles with low leverage [as expected for IT1, IT2, and other hip flexors even in early theropods; e.g., (*16*)] can produce fast, powerful “high-geared” motions (*29*). Thus, we infer that the ability of the hindlimb to be protracted during swing phase phylogenetically “led” the overall reduction of capacity to retract it during stance phase because larger hip flexor muscles with lower leverage preceded the evolution of smaller hip extensors with lower leverage. To our knowledge, this is a new observation that illuminates how hip-based locomotion may have started to reduce.

In addition, however, most hip extensor muscles unusually had peak extensor moment arms around the nodes for Averostra/Avetheropoda. Visual inspection of the models (Fig. 1 and fig. S1) suggests that this was a real outcome of their morphology, combining a caudally extended postacetabular ilium with more distal MTU insertions (if on the femur). Rather than simple, gradual, or stepwise de-emphasis of hip motion during locomotion on the bird-line, we infer from our data that the caudofemoral MTUs of early, large (Jurassic) averostran theropods may have been relatively “low-geared”—large muscles [evidenced by the robust ilium, tail, and fourth trochanter; (*4*, *5*)] with large moment arms, capable of producing large moments for femoral retraction. This specialization does not seem to be simply allometric, as a craniad shift of the body’s CoM also approximately coincides with this region of the phylogeny (*12*), suggesting altered locomotor biomechanics due to underlying morphofunctional changes that cannot be described as allometric in nature. No such specializations are evident from our data for the large-bodied *Plateosaurus* or *Tyrannosaurus*, either, which also contradicts expectations from simple allometry. A similar but generally smaller peak of moment arms happened for several hip extensors of Phasianidae, reflecting the major musculoskeletal and presumed functional changes between Avialae-Aves that strongly reduced hip-based locomotion (*1*–*7*, *9*, *12*, *16*, *21*, *22*, *26*–*34*).

The CFL and CFB were not the only important hip extensors in early bird-line archosaurs. Expected muscle sizes [e.g., (*16*, *23*)] and large estimated moment arms for the ILFB, ADD2, FTE, and FTI3 (and, to a lesser degree, ADD1 and IT3) support the inference that these were critical antigravity and limb retractor muscles in most archosaurs, including early averostran theropods, as they remain in birds (*28*, *33*). Regardless, despite expansion of the postacetabular iliac blade or ischium from which the muscles originated (*5*), no overall trends in hip extensor leverage were found for ILFB, ADD1, ADD2, FTI3, or IT3; yet, muscle sizes likely changed to bring about functional transformations via enhanced force- and thus moment-generating capacities (*16*). This pattern would have allowed these muscles to exert hip extensor moments for weight support or for behaviors not requiring rapid hip motion or high power, such as standing or during slower locomotion [i.e., strut-like function as per (*33*)]. Such supportive specializations would also be beneficial for balancing a more cranially positioned CoM (incurring greater hip joint moment to be balanced) in later theropods relying more on knee-based locomotion, so we speculate that the bird-line co-opted them for this compatible role.

### Transformations of knee flexion/extension (Hypothesis 2)

From our estimates of the evolution of muscle moment arms about the knee, we found that (absolute value) knee flexor leverage mostly decreased in Neotheropoda (Figs. 6 and 8), or overall about −28% from Archosauria to Phasianidae (data file S4). Knee extensor leverage, however, increased (+26% in Neotheropoda and +36% overall across the bird-line; data file S4), contributing to the decreased knee flexor/extensor ratio (Fig. 8). Even quantitatively, then, these changes mirror those of the hip extensor/flexor ratios, which is an exciting congruence in our study’s major results. This observation is a good match for our second hypothesis and supports the inference that stance phase knee flexion arcs (knee-based locomotion) generally increased across theropod dinosaurs. Sensitivity analysis of the branch length assumptions in the phylogeny provided robust secondary support for this inference (fig. S2). In addition, again, we found peak normalized moment arms around the Averostra/Avetheropoda nodes for several knee extensors, strengthening the conclusion that these large early theropods had unusually specialized ways of locomoting (see summary below).

For knee extensors, the changes can be attributed to two morphological transformations: first, the enlargement of the tibial crest in theropod dinosaurs, moving the muscles’ lines of action further cranially from the knee joint center (*35*); second, the ornithuran (i.e., near-avian) pattern of forming even larger, and multiple, cnemial crests and a patella (*21*, *34*). Such enhanced extensor capacity at the knee is consistent with the inference that the final adoption of knee-based locomotion occurred deep within Avialae, but before Phasianidae (*2*). Enlarged knee extensor moment arms would also likely promote the stance phase “antigravity” strut/brake functions that the FMTI/FMTE and IT2 and IT3 muscles seem to have in extant birds (*33*).

In living birds, hip extensor–knee flexor MTUs such as the hamstring ILFB and FTE/flexor cruris act mainly as motors (generating power), in antagonistic or neutral action relative to knee extensor MTUs, which act as brakes or struts (negative or zero power) during stance (*33*). Gatesy (*6*) showed that knee (and hip) excursion arcs increased with speed in running birds (versus walking), so more motor-like functions might be expected of hamstring MTUs in these behaviors. One way in which a change into increased motor-like functions of knee flexors (and corresponding behavioral changes) could have happened (all else being equal) is that knee flexors simply became larger from Averostra onward. As noted above, anatomical evolution supports this speculation: The caudal iliac blade that served as the origin for some of the major flexor cruris group of muscles expanded both caudally and dorsoventrally in Neotheropoda (*5*, *16*). Alternatively or in addition, the advance of the activation onset of ILFB from swing phase (as in Sauria plesiomorphically) into stance phase [as in extant birds; (*7*, *36*)] may have begun to evolve in neotheropods. This advance would similarly have enhanced knee flexor MTU moment-generating capacity during stance phase. Either or both changes would have produced somewhat more knee-based locomotion in Neotheropoda or later. While literature on the shift from hip- to knee-based locomotion on the bird-line often has emphasized kinematic changes such as joint ranges of motion (RoM) [e.g., (*6*, *9*)], these changes must have been produced by kinetic mechanisms such as musculotendinous moments, hence our logic here. These changes we propose here emphasize the complex nature of sequences of changes in knee MTU form, function, and control that sequentially produced more components of the derived knee-based locomotor mechanism seen in extant birds.

### Transformations of hip medial rotation/abduction (Hypothesis 3)

Our estimated evolutionary trends in moment arms for hip LAR are also highly consistent with our third hypothesis, with numerous increases between Neotheropoda and Phasianidae (Figs. 5 and 8). Summed (absolute value) medial rotator moment arms increased an impressive +146% across the entire bird-line, whereas abductors increased only by +24% (data file S5). Again, sensitivity analysis of the branch length assumptions in the phylogeny gave ancillary support to this third hypothesis (fig. S2). The reduction of the ratio of medial LAR versus abductor leverage was contributed to both by increases of medial rotation at Neotheropoda (+67%) and Phasianidae (+64%) and by decreases of abduction from the Coelurosauria to Avialae nodes (~−20% per node; data file S5). These changes can be understood by first considering control of stance phase frontal plane moments in extant archosaurs, following up on points from Introduction, which we review here.

In extant birds, lateral LAR hip moments induced by loading of the body and limb under gravity and inertia (e.g., the GRF) are balanced by medial LAR hip moments from activation of the ITC and *M. iliotrochanterici medialis* (*7*), homologs of the PIFI2 in Crocodylia (*5*, *23*, *37*). MTUs controlling LAR in birds seem to act as zero-length-change struts rather than springs, brakes, or motors (*33*). In extant Crocodylia, the pes is generally placed lateral to the hip, and so the GRF exerts a hip abductor moment that is thought to be mainly countered by activation of the ADD1 and ADD2, although many stance phase limb muscles that originate ventral to the hip are likely to also contribute useful hip adductor moments (*2*). The roles of crocodylian hip MTUs in LAR are more poorly understood than in birds, and it is not yet clear if hip adductors act as struts (analogous to LAR-controlling MTUs in birds). Given the gradual abduction of the femur throughout stance phase in Crocodylia (*38*) and the more parallel-fibered, uniarticular, or short-tendoned hip extensors such as ADD1 and ADD2 and the flexor cruris muscles, these stance-phase active muscles might act more as brakes, controlling hip abduction while actively lengthening. Ancestral archosaurs may have used the same mechanism (*2*).

The crocodylian *M. iliofemoralis* (IF), homolog of the dinosauromorph/avian ITC, is a hip abductor and is active during swing phase (not stance phase like the ITC) to lift the limb (*2*, *38*), presumably performing a motor-like function. Transformation of the IF/ITC from swing phase abductor to stance phase medial rotator (and perhaps from motor to strut function) is thought to have occurred in two phases. First, because bipeds place their pes medial to the hip (near the sagittal midline) to maintain balance, the GRF exerts an adductor rather than abductor moment about the hip. As the major hip abductor, stance phase IF/ITC activity (perhaps transformation into a strut-like function as an abductor) is therefore hypothesized to have evolved within Dinosauromorpha as a necessary step in the evolution of dinosaur bipedalism (*2*). Second, expansion of the IF/ITC’s origin on the iliac blade in dinosaurs is thought to have shifted the MTU’s line of action cranially, transforming it from a hip abductor to a medial rotator [but maintaining the strut-like function; (*33*)]. Previous work suggested that this pattern was essentially complete by Eumaniraptora (*2*, *27*). Our findings illuminate how these changes in neuromuscular control of frontal plane balance may have evolved on the bird-line, unifying data from experimental and simulation-based studies with models of MTU leverage in extinct and extant archosaurs.

We also found that most (if not all) MTUs crossing the hip would have exerted some frontal-plane moments about the hip, making control of mediolateral balance via hip LAR complex. As per the paragraph above [also see Introduction and (*2*)], the adductors ADD1 and ADD2 are thought to have ancestrally opposed gravity-induced hip abduction during stance phase whereas the avian ITC, a derivative of the ancestral IF, switched from swing phase to stance phase activity in bipedal Dinosauromorpha to oppose gravity-induced hip adduction. Avian hip adductor homologs have preserved their ancestral activity during stance phase (*7*). Across archosaurian evolution (Figs. 3 to 5), we found that the ADD1 and ADD2 remained hip extensors and adductors (except in Phasianidae), but very weak in hip LAR, whereas the ITC remained hip flexors (weak), abductors, and medial rotators. Thus, these two muscle groups might outwardly, from EMG data, seem to have maintained antagonism, but their moment arm values tell another story. The adductors and the ITC (with partial homolog *M. iliofemoralis externus*; IFE) exhibited a synchronized shift away from ancestrally large antagonistic moment arms for hip adduction/abduction (respectively) (Fig. 4). We infer this shift to reflect an early archosaurian reliance on antagonism of the ADD (stance phase) versus IF (swing phase) muscle groups that was later disrupted (e.g., within averostran theropods). This disruption in later theropods arose from the two muscle groups eventually evolving divergent primary leverages during stance phase, eliminating the opposition of adduction versus abduction. The ADD1 and ADD2 emphasized larger moment arms for hip extension rather than adduction (Fig. 3), whereas the ITC (and IFE) emphasized larger moment arms for hip medial LAR rather than abduction (Fig. 5); also see further below. A caveat for our LAR results is that femur orientation was maintained vertical when computing mean MTU moment arms; future analyses should explore covariation between increasingly horizontal femora and LAR mechanics across the bird-line.

Interestingly, the ~+100% increase of hip abductor leverage at Dinosauromorpha (versus Archosauria) coincides with the presumed origin of bipedalism (*2*–*5*), as exemplified by our model of *Marasuchus*, and corroborating the hypothesis that transformations in hip abductor form, function, and motor control evolved in this ancestor (*2*). We did not originally predict this, but it is an exciting outcome of our analyses, deserving further inquiry with additional models of avemetatarsalians and pseudosuchians. This outcome was absent in our extreme model of branch lengths (fig. S2), so we view it cautiously, although our original model was more plausible.

In addition to the above shifts of ITC roles from abduction to medial LAR (Figs. 4 and 5), medial LAR leverage for the PIFI2, part of which acts as a stance phase medial rotator in extant birds (*33*, *37*), also showed a less extreme, and roughly concurrent, trend of increase (roughly doubling; also mirroring its hip flexor leverage decrease). It is interesting that the ITC and PIFI2 (with PIFI1) are among the few muscles to consistently have medial LAR moment arms—almost all of the other hip muscles out of the 20 modeled acted more in lateral LAR, and some of these are expected to have been among the largest hip muscles (e.g., CFL, CFB, ILFB, and IT3). As these represent the majority of the hip muscles that can be conservatively assumed to have been active during stance phase [e.g., not PIFE1 to PIFE3; ancestrally a swing phase group; (*2*)], it is difficult to see how lateral LAR moments would not have been incurred by muscles acting around the hip during stance phase. Most of these lateral LAR moment arms were small (e.g., ~0.040 for CFL and CFB versus 0.090 mean for PIFI2), so antagonistic moments might not have been so large, and activity of the PIFI2 in ancestral Archosauria should have been concentrated more in swing than in stance phase (*7*, *37*). This problem of antagonism of LAR during stance phase, however, had not previously been anticipated and may be a key part of the torsionally loaded, horizontal femur characteristic of extant avian knee-based locomotion (*4*, *5*, *7*, *19*, *30*, *31*).

Considering the above patterns, our finding of early increases of overall medial LAR moment arms either might be incidental or might reflect a shift in, for example, some activation timing of PIFI2 (i.e., homolog of the avian *M. iliotrochantericus medialis*; Table 1) to stance phase, satisfying the biomechanical requirement to balance hip lateral LAR moments caused by activity in other hip muscles, perhaps even in bipedal Dinosauromorpha. The latter inference would not necessarily, however, involve more horizontally oriented femora changing the relationship between the GRF and the hip from more adduction into more lateral LAR, which was a change likely more characteristic of later theropods (e.g., Averostra gradually onward), yet it might be one additional evolutionary step preceding such postural changes. However, as passive tissues (e.g., bones and ligaments) could resist hip abduction (*32*, *33*), these could even have played a simultaneous role in resisting hip lateral LAR during stance phase, reducing muscular demands on medial rotators. The above-described series of transformations in the relationship between tissues controlling LAR is a novel idea deserving more inquiry.

**Table 1:** Major (35+) hindlimb muscles of Archosauria, with names for homologous muscles in Crocodylia and Aves/Phasianidae and corresponding abbreviations used here. Suspected main “actions” are listed. Moment arms about toe joints were not studied so muscles such as the EDB and FDB were not analyzed in this study. Different homologies of the EDL/TA and FDL/FHL have been proposed recently (*50*), but, as their moment arms were similar, these would not fundamentally alter our results or conclusions.

Muscles (Crocodylia)	Abbreviation	Muscles (Aves/Phasianidae)	“Main” action
*M. caudofemoralis longus*	CFL	*M. caudofemoralis pars caudalis*	Hip extensor
*M. caudofemoralis brevis*	CFB	*M. caudofemoralis pars pelvica*	Hip extensor
*M. iliofibularis*	ILFB	*M. iliofibularis*	Hip extensor/knee flexor
*M. adductor femoris 1*	ADD1	*M. puboischiofemoralis medialis*	Hip adductor/hip extensor
*M. adductor femoris 2*	ADD2	*M. puboischiofemoralis lateralis*	Hip adductor/hip extensor
*M. flexor tibialis internus 1*	**FTI1**	**Lost between Avialae-Aves**	Hip extensor/knee flexor
*M. flexor tibialis internus 2*	**FTI2**	**Lost in Dinosauromorpha—not** **reconstructed**	Hip extensor/knee flexor
*M. flexor tibialis internus 3*	FTI3	*M. flexor cruris medialis*	Hip extensor/knee flexor
*M. flexor tibialis internus 4*	**FTI4**	**State in fossil taxa ambiguous—** **not reconstructed**	Hip extensor/knee flexor
*M. flexor tibialis externus*	FTE	*M. flexor cruris lateralis pars pelvica*	Hip extensor/knee flexor
**State in fossil taxa ambiguous—not** **reconstructed**	-	*M. flexor cruris lateralis pars accessoria*	Hip extensor
*M. ischiotrochantericus*	ISTR	*M. ischiofemoralis*	Hip extensor/hip lateral rotator
*M. ambiens 1*	AMB1	*M. ambiens*	Hip flexor/knee extensor
*M. ambiens 2*	**AMB2**	**Absent; presumed autapomorphy** **of Crocodylia**	Hip flexor/knee extensor
*M. iliotibialis 1*	IT1	*M. iliotibialis cranialis*	Hip flexor/knee extensor
*M. iliotibialis 2*	IT2	*M. iliotibialis lateralis pars preacetabularis*	Hip flexor/knee extensor
*M. iliotibialis 3*	IT3	*M. iliotibialis lateralis pars postacetabularis*	Hip extensor/knee extensor
*M. puboischiofemoralis externus 1*	PIFE1	*M. obturatorius lateralis*	Hip flexor/hip lateral rotator
*M. puboischiofemoralis externus 2*	PIFE2	*M. obturatorius medialis*	Hip flexor/hip lateral rotator
*M. puboischiofemoralis externus 3*	**PIFE3**	**Lost between Avialae-Aves**	Hip flexor/hip lateral rotator
*M. puboischiofemoralis internus 1*	PIFI1	*M. iliofemoralis internus*	Hip flexor/hip medial rotator
*M. puboischiofemoralis internus 2*	PIFI2	*M. iliotrochantericus cranialis*	Hip flexor/hip medial rotator
**State in fossil taxa ambiguous—not** **reconstructed**	-	*M. iliotrochantericus medius*	Hip flexor/hip medial rotator
*M. iliofemoralis*	IF	*M. iliofemoralis externus*	Hip abductor
**Evolved in Dinosauromorpha**	**ITC**	*M. iliotrochantericus caudalis*	Hip abductor/hip medial rotator
*M. femorotibialis externus*	FMTE	*M. femorotibialis lateralis*	Knee extensor
**State in fossil taxa ambiguous—not** **reconstructed**	-	*M. femorotibialis intermedius*	Knee extensor
*M. femorotibialis internus*	FMTI	*M. femorotibialis medialis*	Knee extensor
*M. gastrocnemius lateralis*	GL	*M. gastrocnemius (pars) lateralis*	Knee flexor/ankle plantarflexor
*M. gastrocnemius medialis*	GM	*M. gastrocnemius (pars) medialis*	Ankle plantarflexor
**State in fossil taxa ambiguous—not** **reconstructed**	-	*M. gastrocnemius (pars) intermedius*	Ankle plantarflexor
*M. extensor digitorum longus*	EDL	**Split into *M. tibialis cranialis*—** ***caput femorale* and *caput tibiale* in** **Aves**	Ankle dorsiflexor (knee extensor?)
*M. extensor hallucis longus*	EHL	*M. extensor hallucis longus*	Ankle dorsiflexor
*M. extensor digitorum brevis*	EDB	**Ancestral TA and EDB fused in** **Aves to form *M. extensor digitorum******longus*; EDB not reconstructed for** **fossils**	Toe extensor
*M. tibialis anterior*	TA		Ankle dorsiflexor
*M. flexor digitorum longus*	FDL	*M. flexor digitorum longus*	Ankle plantarflexor/toe flexor
*M. flexor hallucis longus*	FHL	*M. flexor hallucis longus*	Ankle plantarflexor
*M. flexor digitorum brevis*	**FDB**	**State in fossil taxa ambiguous—** **not reconstructed**	Toe flexor
*M. pronator profundus*	**PP**	**Lost in Dinosauromorpha—not** **reconstructed**	Tibiofibular rotator
*M. interosseus cruris*	**IC**	**Lost in Neotheropoda (only** ***M. popliteus* part left)—not** **reconstructed**	Tibiofibular rotator
*M. fibularis longus*	FL	*M. fibularis longus*	Ankle plantarflexor
*M. fibularis brevis*	FB	*M. fibularis brevis*	Ankle dorsiflexor?

Perhaps as importantly, the only major evolutionary trend for lateral LAR hip muscles was that the PIFE1 and PIFE2 MTUs had consistently increasing moment arms (Fig. 5) across the phylogeny (taking over from the PIFE3 as it was reduced and lost, and aiding the ISTR). These are expected to have been “swing phase” muscles involved in control of mediolateral balance based on LAR about the hip joint (*2*), an ability our results indicate should have increased across the bird-line. All other muscles with lateral LAR actions showed either negligible trends or decreasing moment arms. Furthermore, essentially all hip adductors decreased their leverage across the bird-line (Fig. 4), nicely reflecting the predicted shift from emphasizing stance phase hip adduction in Archosauria to abduction in Dinosauromorpha and then LAR control along the bird-line (*2*). As a consequence, extant birds were left with weak hip adductors replaced by passive mechanisms of adduction such as the antitrochanter and pelvic ligaments (*32*, *33*).

### Transformations of ankle function across the bird-line

Last, we found that moment arms for ankle extension increased slightly across the bird-line (Fig. 7 and data file S6), despite the loss of the calcaneal tuber in dinosaurs, an increase contributed to by the evolution of the hypotarsus and enlarged tibiotarsal condyles in birds (*21*). While there were some reductions of this leverage, and evolutionary patterns were noisy (variable) as a result, the nodes Averostra, Avetheropoda, and Avialae each had ~25% contributions to the overall 14% increase of leverage across the bird-line.

In Crocodylia, ankle plantarflexion contributes to propulsion during later stance (*38*), whereas in extant birds, the ankle remains relatively static, supplying little (or no) net propulsive power during stance (*30*, *33*). Larger moment arms would have facilitated use of the elongate metatarsus as a zero-power strut, as observed in extant birds (*33*), rather than as a propulsive lever, as inferred in extant Crocodylia. We infer this facilitation because a quasi-static strut resisting gravitational and inertial loads throughout stance phase may require larger moments than the smaller ground-reaction (and limb) forces at the end of stance phase when propulsion is concentrated. Our results of increasing ankle extensor moment arms from approximately Averostra to Avialae roughly coincide with (i) unusually deep footprints hinting that early theropods (e.g., Averostra) did not orient their metatarsus as vertically throughout stance phase as in extant birds, probably sweeping their ankle joint through a considerable, non-strut-like RoM (*39*) and (ii) elongation of the metatarsus around the base of Avialae (*18*–*20*). Together, these observations support the interpretation of the ankle of Avialae as more erect and “strut-like” than that of their earlier dinosaurian ancestors. We therefore suggest that the observed divergence in ankle use between extant archosaurs may have evolved by Avialae, with a gradual change continuing through to crown group birds (*21*), although some early changes in the mechanics of support already existed in Triassic/Jurassic theropods (e.g., Averostra). A caveat is that body size changes [e.g., miniaturization near Avialae; (*12*) and Fig. 1] may have altered ankle mechanics in more complex ways.

### Overall changes of pelvic limb muscle form and function

As discussed above, the bird-line’s transition from hip-based to knee-based locomotion involved a general reduction of hip extension arcs and increase in knee flexion arcs (*4*, *6*). Discussion of hip and knee flexion/extension as separate entities is complicated by the numerous biarticular muscles involved—several large hip extensors are also knee flexors (e.g., ILFB and flexor cruris), and some major hip flexors (e.g., IT1 and IT2) are also knee extensors (see also the “Assumptions” section in Materials and Methods). Overall, our data addressing Hypotheses 1 to 3 support de-emphasis of hip-based locomotion, shifting into greater knee-based locomotion, from Neotheropoda onward [as suggested by the anatomical evolution of the tail and general body shape; (*4*, *10*–*12*)].

We have found unexpected support for unique specializations of muscular leverages in early theropod dinosaurs (Neotheropoda-Avetheropoda), such as peaks for moment arms of major hip extensors (ILFB, ADD2, and FTI1) and flexors (PIFE1 and PIFE3), hip abductors (CFB, ILFB, and IT3), hip medial (FTI1 and FTE) and lateral (PIFE3) rotators, and knee flexors and extensors. The specializations in Averostra-Avetheropoda are concomitant with gradually increasing ancestral body sizes, a size-related shift of limb proportions toward less cursorial morphology [(*18*); longer femur, shorter lower limb], and a strong craniad shift of the whole-body CoM (*12*), further indicating important alterations of locomotor biomechanics including reduced athleticism, as well as a more macropredatory ecology (*40*). Furthermore, the changes coincide with key morphofunctional transformations including twisting of the femoral head to orient medially [(*5*); altering hip LAR moment arms as shown here] and more mobile hip joint articulations, unlike those in Neotheropoda (*17*). This evidence suggests that early theropods evolved through a series of ancestral states of musculoskeletal function quite unlike those of early archosaurs, coelurosaurs, or birds, with these states being lost later on the bird-line. The series was part of the shift from hip- to knee-based locomotion but involved apomorphic intermediate states that do not fit simply into that continuum. Thus, the bird-line involved unique evolutionary stages more complex than merely a gradual series of more “bird-like” hindlimb functions [e.g., (*9*)]. Rather, these ancestral stages included small, cursorial, highly parasagittally constrained Neotheropoda (*3*, *16*, *17*), a sequence of large Averostra-Avetheropoda, and then the increasingly miniaturized, knee-driven Coelurosauria including Avialae, each with apomorphic limb functions lost or radically transformed in later descendants.

As we discuss in Materials and Methods, our study has key limitations. The quantitative moment arm results from individual models, transformed into evolutionary ACEs, have many sensitivities from anatomical accuracy to sample size to phylogenetic branch lengths. The quantitative nature of our results (including the crude metric of summed, mean moment arms) are the best approximations feasible at present given the vagaries of the fossil record. Yet, we contend that these are better than purely qualitative functional anatomy, which had not previously identified many of the major changes in musculoskeletal function we have here, such as the apparent specializations of early, large theropods. Our sensitivity analysis using very different (“punctuated model”) branch lengths (fig. S2) gave strong support for our conclusions on Hypotheses 2 and 3, alleviating concerns about the potential influence of branch lengths.

Our study has shown how a major transition in terrestrial locomotion evolved across the archosaurian bird-line, involving anatomical changes that altered the biomechanical actions (moment arms or leverages) of many pelvic limb muscles. Advancing from useful qualitative analyses of a few muscles [e.g., (*2*, *4*)], we have quantified how the actions of 35 hindlimb muscles evolved across ~250 million years, an unprecedented undertaking in any taxon to date, to our knowledge. We have integrated these biomechanical data with other available data from anatomy, ichnology, and neuromuscular control in a new synthesis. Researchers could use our open dataset to test other questions such as how apomorphic muscle actions evolved in relation to extreme morphological changes on deeply nested branches, such as placing specialized pseudosuchians, ornithischians [e.g., (*38*)], sauropodomorphs, maniraptoran subclades (e.g., therizinosauroids), and avialans into the general archosaurian phylogenetic framework we have established. Future steps could involve better estimates of the capacities of muscles to produce forces and length changes as well as more dynamic analyses [e.g., (*16*, *22*, *33*)] of locomotion, all of which could be implemented by later studies using the three-dimensional (3D) computer models provided here.

## MATERIALS AND METHODS

### Specimens

Musculoskeletal models were created for the 13 taxa shown in Fig. 1 (enlarged views provided in fig. S1). Details of specimen numbers, completeness, and overall body size are in data file S1. The range of taxa used in this study was chosen based on accessibility for scanning, the completeness of the hindlimb skeleton, and their suitability for representing general morphological disparity along the archosaur bird-line in sufficient resolution. Models derived from the whole-body modeling analyses of (*12*) were used, except that badly crushed, more 2D remains deemed too unreliable for moment arm analyses—particularly early birds—were omitted here. Following convention, galliform Phasianidae (red-necked junglefowl *Gallus gallus* and common pheasant *Phasanius colchicus*) were used to represent crown group birds, justified by their small body size and more plesiomorphic, cursorial morphology (*2*–*7*). These two taxa split up the long branch for Phasianidae divergence and so were deemed superior to just one taxon or to the anatomy of highly specialized birds such as ostriches (*33*).

### Estimating moment arms

MTU moment arms in the hindlimbs of fossil bird-line archosaurs were calculated from the 13 computer models using Software for Interactive Musculoskeletal Modeling (SIMM) version 6.0 (Musculographics Inc., Santa Rosa, CA, USA). Estimation of MTU moment arms in that software via the “virtual work” method (*24*, *25*) requires estimation of the location of the rotation center and axes for each joint and estimation of the MTU line of action (the muscle path as it crosses the joint). Quantification of these data involved three stages: (i) acquisition of skeletal geometry, (ii) estimation of joint rotation centers and axes, and (iii) estimation of MTU paths.

#### *Digitization of skeletal material*

Fossil bone geometry was captured variously using handheld (Polhemus FastScan COBRA, https://polhemus.com/scanning-digitizing/fastscan/) and mounted (Konica Minolta VIVID 910, www.konicaminolta.com) laser surface scanners and point digitizers (Freepoint 3D Sonic Digitizer, GTCO CalComp, Columbia, MD, USA). Nonfossil bone geometry was acquired using a computerized tomography scanner (GE Picker PQ5000). All digitized skeletons were processed using computer-aided design software (Polhemus FastScan and Blender) and reduced to ASC/STL format polygonal mesh files. A complete, right pelvic limb was assembled (mirroring left elements if necessary) for each taxon (data file S1).

#### *Estimation of pelvic limb joint rotation centers*

Joint rotation centers were estimated from the articular surfaces of the digitized bones using a simple geometric method. Hip joints were reconstructed as three DoF ball-and-socket joints, with rotation centers for all DoFs (flexion/extension [FLEXEX], abduction/adduction [ABAD], and long-axis rotation [LAR]) located at the superimposed geometric centers of spheres (Fig. 2A) manually fitted around both the head of the femur (femoral segment) and the internal surfaces of the acetabulum (pelvic segment). The knee and ankle were reconstructed as single DoF (FLEXEX) revolute joints, with the centers located at the long-axis midpoint of a cylinder fitted around the distal femoral condyles (Fig. 2A) and the compound articular surface of the astragalus and calcaneum, respectively.

The joints of extant animals are seldom so constrained. Complex, coupled rotations and translations about compound, mobile axes dependent on the interactions of ligament and joint capsule anatomy, articular surface geometry, and loading regime are probably the norm (*17*, *30*–*33*). Our assumptions of fixed rotation centers, located approximately at the geometric centers of the articular surfaces, and a single DoF for the knee and ankle, are simplifications that emphasize osteological data. However, links between skeletal anatomy and joint function are not currently well understood for even extant crocodylian or avian joints, although recent studies have made progress [e.g., (*17*, *41*)]. Attempting more detailed reconstructions of joint motion envelopes in fossil archosaurs therefore invokes an excessively large list of assumptions for the purposes of this study. The simple, geometrically determined joint centers and DoFs used here, while highly likely to be inaccurate to varying degrees, are based on simple assumptions and are easily repeatable across our study sample.

We used “right-handed” coordinate systems for our joints as follows. FLEXEX axes for all joints faced (right) laterally, perpendicular to the sagittal plane. Positive FLEXEX rotation flexed the hip, extended the knee, and (dorsi)flexed the ankle, and negative FLEXEX rotated in the opposite direction (Fig. 2B). For the hip joint, the ABAD axis faced cranially, perpendicular to the frontal plane. Positive ABAD rotation adducted the hip, and negative ABAD rotation abducted it. When the femur was vertical, positive LAR was medial/internal rotation; negative LAR was lateral/external rotation. Joints rotated in the order FLEXEX-ABAD-LAR; there were no translations. Zero degrees of rotation for each joint DoF aligned all joint centers vertically, so that the limb was fully extended ventrally in a nonbiological “reference pose” (Fig. 2B). This approach blended prior ones (*26*–*29*).

In life, the bones of the limb joints are assumed to have been separated by some thickness of joint cartilage, menisci, and other arthrological structures including missing (unmodeled) distal tarsal bones (*17*, *26*, *27*, *41*). To represent this, the joints were translated apart by a fixed percentage of the proximal segment’s length (5% in the knee and 7.5% in the ankle), whereas the hip joint was in the middle of the acetabulum. This linear transformation presumably had small influences on moment arms and, in any case, maintained consistency across models.

#### *Reconstruction of pelvic limb MTUs and lines of action*

The extant outgroups to extinct dinosauromorphs (living birds and crocodiles) exhibit MTUs that are expected in bird-line archosaurs, including their osteological correlates and their likely origins and insertions [Fig. 2, C to E, and Table 1; modified from (*2*, *16*, *35*)]. The full list of 35 MTUs was reconstructed for all taxa except as indicated. Acronyms used for all muscle names are in Table 1.

In living animals, MTU lines of action rarely are straight vectors between origin and insertion, instead deflecting around bones and other limb tissues. To reconstruct more plausible lines of action, we added “via points” to our MTU path vectors, and cylindrical, ellipsoidal, or spheroidal “wrapping surfaces” (around which the software automatically deforms MTU paths on contact) to represent underlying muscle or bone in our models (Fig. 2, C to E).

### Joint ranges of motion

For comparability, moment arms were output from the software for the same joint RoM in all taxa. Although we report moment arms for each of the three DoFs for the hip (FLEXEX, ABAD, and LAR), allowing each of them to vary simultaneously within a wide range creates a problematically large (and difficult to interpret; hyperdimensional—i.e., plotting 3D moment arms against each of three DoFs) dataset. Instead, in the default initial pose (and for FLEXEX RoM), we locked the hip joint at −15° of ABAD to allow for limb clearance of the abdomen, and 0° of LAR to align joint flexion/extension with the assumed direction of travel (an admitted simplification). MTU moment arms were then estimated for FLEXEX, ABAD, and LAR over the following RoM for each joint: hip joint −45° (extension) to 65° (flexion), −45° to 0° of ABAD and −30° (medial/internal) to +30° (lateral/external) of LAR, knee joint −90° (flexion) to 10° (extension), and ankle joint 0° (extension/plantarflexion) to 90° ([dorsi]flexion). The (third) metatarsophalangeal joint was fixed at 0°; i.e., metatarsus perpendicular to pes (this incurred only trivial issues for comparisons with the actually plantigrade crocodile). While such large RoMs are likely to exceed those used in vivo by some taxa (especially for the hip), they were chosen to encompass the maximum plausible range of flexion/extension for locomotion. Models are provided on Figshare (https://figshare.com/articles/dataset/Archive_of_all_13_musculoskeletal_models/12776084).

### Moment arm data normalization

Each taxon’s moment arms for each MTU around each DoF of a joint were automatically calculated in SIMM software through the entire RoM, exported as text files and reduced to a mean value for each MTU (and DoF) via a custom script in GNU Octave open source software (www.gnu.org/software/octave/), used for all further processing. Data for MTUs split into two subheads (PIFI2 for Crocodylia, IT2 in general, and ITC for Dinosauria) were averaged. Mean moment arms were normalized to allow direct comparison between taxa. There are expected allometric changes of moment arms relative to body mass [e.g., (*13*, *40*)], so body mass^0.33^ was deemed undesirable for normalization. Instead, mean MTU moment arms were normalized by dividing them by relative segment lengths (femur for hip, tibiotarsus for knee, and tarsometatarsus for ankle MTUs). Although there are phylogenetic (and allometric) changes to segment lengths (*18*–*20*), they at least relate more directly to the size-related musculoskeletal morphology of their joints (and hence moment arms) than linearized body mass does. Figure 1 includes body masses of modeled specimens for reference [from (*12*)].

### Phylogenetic optimization of moment arm data

We output the mean normalized moment arm data from GNU Octave as .CSV files (one per MTU and corresponding DoFs) for phylogenetic trait mapping (optimization). Because all taxa in this study were terminal taxa, representing to some extent specialized side branches along the phylogeny, their hindlimb anatomy was likely to differ somewhat from that present in the ancestral nodes linking archosaurs together. Therefore, we had to estimate the moment arms present at the internal nodes of the archosaur phylogeny to understand how moment arms and their ratios have changed during the transition from hip- to knee-based locomotion. To achieve this estimation, maximum likelihood ACEs for mean normalized moment arms were calculated for each ancestral node, using the packages ape (*42*) and phytools [(*43*); https://github.com/liamrevell/phytools/] for R 3.3.2 software (R Foundation for Statistical Computing, Vienna, Austria; www.R-project.org), as follows.

We reconstructed ACEs via the fastAnc function in the R package phytools [(*43*); www.phytools.org/static.help/fastAnc.html]. In addition to mean moment arm data for each sampled taxon, fastAnc requires a cladogram, with calibrated branch lengths, describing the phylogenetic relationship between taxa. This phylogeny (Fig. 1) was constructed using a simplified, high-level phylogenetic framework [based on (*44*–*47*; also data file S2)]. Branch lengths (Fig. 1) were set to the time in million years separating nodes, taken from fossil and molecular data on divergence times or estimated from the ages of fossils from earliest known clade members. Node and taxon dates are in data file S2. “Averostra” here refers to the unnamed node connecting *Dilophosaurus* + Avetheropoda although Averostra normally indicates [Tetanurae + Ceratosauria], a less inclusive clade. As our two avian taxa are both phasianid galliforms, we use Phasianidae as a proxy here for Aves (Fig. 1) pending future biomechanical analyses of non-galliform crown birds.

### Statistical analysis

ACEs from fastAnc were output as the independent contrast state values for each ancestral node and exported from R as .CSV files of results (ancestral moment arms versus nodes across the phylogeny). We then further processed ACE values individually for each MTU and DoF via a GNU Octave script into final .CSV files (i.e., 3 for hip, 1 for knee, and 1 for ankle). These processed values were then used to plot changes in moment arm values for each MTU across the phylogeny’s internal nodes. To quantify ratios of moment arms (for Hypotheses 1 to 3) in a simple way, we summed the absolute values of all MTU moment arm ACEs acting in a particular direction for a DoF (e.g., hip extensors) to produce one summed (positive) ACE value for each node. As fastAnc produces 95% confidence intervals around the contrast states for ACE values, we considered these in our results and conclusions. However, for simplicity in testing our Hypotheses 1 to 3, we a priori set a threshold of ≥20% change in the ACEs of ratios of summed moment arm between any two successive nodes on the phylogeny (Fig. 1) as a sufficiently reliable result, but we also deemed ≥10% internodal changes as worthy of discussion.

### Assumptions

“Muscle” and MTU are used interchangeably here, but, as per (*33*), only gross MTU functions can be broached (at best) with our data; muscle fiber-level mechanics cannot be distinguished from tendon or other passive tissue contributions. Our analyses of moment arms exclude multiarticularity (only focusing on 1 DoF at a time; no coordinated RoMs of joints as would happen in vivo) and are entirely static (i.e., omitting complex intersegmental dynamics). Furthermore, our analyses could not consider gravity or inertial forces, are nonphysiological (unable to consider muscle force-length, force-velocity, or other parameters known to have complex interactions with MTU moment arms), and make other such necessary assumptions (*24*, *25*, *26*, *29*). We maintain, however, that the “actions” of muscles (anatomically implied functions) focused on here have some biological reality that is informative about higher-level organismal behavior and apply what we feel is appropriate caution and addition of relevant data in Discussion where warranted and feasible. Thereby, we conduct an original analysis of the evolution of 35 hindlimb MTU leverages across the bird-line that will inspire and enable future revision, reuse, and replication. Furthermore, we built all the models following the same procedure and assumptions. Such consistency is an advantage for our comparative analyses, which we hope will thereby be better able to identify relative changes in function qualitatively, even if quantitative precision is not ideal despite our unique provision of statistical analyses [cf. (*28*)].

Our phylogenetic algorithms required that data from the ITC and IFE moment arms were optimized to the Archosauria node as well, which should be viewed with caution because these muscles did not diverge until Dinosauromorpha (*2*). Yet, this was similar to our assumption (see moment arm normalization above) that large muscles may be split into two heads to allow for heterogeneous mechanical function, even if anatomically not distinct. *Crocodylus* was used as the sole representative of Pseudosuchia, following the inferences of (*2*) that its general muscle anatomy and actions are comparable to, and largely homologous to, ancestral Archosauria [supported by other studies, e.g., (*28*)]. This comparability is, despite its variable limb posture, secondarily amphibious habits, mobile pubis, and other traits, unlikely to directly affect our results for MTU moment arms. As we modeled it in the same default limb postures as other archosaurs, this maximized comparability. An additional factor that could affect our estimated ancestral values of moment arms is our phylogeny’s branch lengths (*48*, *49*); however, robustly testing the influence of branch lengths upon models ideally requires a sample size beyond the scope of the present study. To provide a preliminary test of the impact of branch length assumptions on our conclusions, we set all branch lengths to 1 (approximating a “punctuated” model of evolution) and reran the analysis under this more extreme scenario of lineage divergence. Similarly, the phylogenetic resolution of our sampling can be built upon in future studies using our open dataset, but we contend that it is sufficient for the questions addressed here. Studies such as (*28*) showed modest general variation in moment arms of other, more deeply nested (and highly apomorphic, corresponding to where moment arms were the most divergent) archosaurian taxa such as ornithischians and *Poposaurus*.

## Supplementary Material

http://advances.sciencemag.org/cgi/content/full/7/12/eabe2778/DC1

Data file S1

Data file S2

Data file S3

Data file S4

Data file S5

Data file S6

Data file S7

Adobe PDF - abe2778_SM.pdf

The evolution of pelvic limb muscle moment arms in bird-line archosaurs

## SUPPLEMENTARY MATERIALS

Supplementary material for this article is available at http://advances.sciencemag.org/cgi/content/full/7/12/eabe2778/DC1

## Appendix

View/request a protocol for this paper from *Bio-protocol*.
