# Supplementary material for: The evolution of pelvic limb muscle moment arms in bird-line archosaurs
Source: Sci Adv. 2021 Mar 19;7(12):eabe2778. doi: 10.1126/sciadv.abe2778 (PMC7978429; doi:10.1126/sciadv.abe2778)
Supplement: http://advances.sciencemag.org/cgi/content/full/7/12/eabe2778/DC1 [file supp_7_12_eabe2778__7.12.eabe2778.DC1.html]

Science Advances | Science AdvancesAAASSearchScience AdvancesMenu

## Supplementary Materials

# The evolution of pelvic limb muscle moment arms in bird-line archosaurs

V. R. Allen, B. M. Kilbourne, J. R. Hutchinson

Download Supplement

**The PDF file includes:**

- Figs. S1 and S2
- Text S1
- Legends for data files S1 to S7

**Other Supplementary Material for this manuscript includes the following:**

- Data file S1
- Data file S2
- Data file S3
- Data file S4
- Data file S5
- Data file S6
- Data file S7

**Files in this Data Supplement:**

- Adobe PDF - abe2778\_SM.pdf
- abe2778\_Data\_file\_S1.xlsx
- abe2778\_Data\_file\_S2.xlsx
- abe2778\_Data\_file\_S3.xlsx
- abe2778\_Data\_file\_S4.xlsx
- abe2778\_Data\_file\_S5.xlsx
- abe2778\_Data\_file\_S6.xlsx
- abe2778\_Data\_file\_S7.zip
