# Supplementary material for: The evolution of pelvic limb muscle moment arms in bird-line archosaurs
Source: Sci Adv. 2021 Mar 19;7(12):eabe2778. doi: 10.1126/sciadv.abe2778 (PMC7978429; doi:10.1126/sciadv.abe2778)
Supplement: http://advances.sciencemag.org/cgi/content/full/7/12/eabe2778/DC1 [file supp_7_12_eabe2778__abe2778_SM.pdf]

Science Advances
AAAS

advances.sciencemag.org/cgi/content/full/7/12/eabe2778/DC1

Supplementary Materials for

The evolution of pelvic limb muscle moment arms in bird-line archosaurs

V. R. Allen, B. M. Kilbourne, J. R. Hutchinson*

*Corresponding author. Email: jhutchinson@rvc.ac.uk

Published 19 March 2021, *Sci. Adv.* **7**, eabe2778 (2021)
DOI: 10.1126/sciadv.abe2778

The PDF file includes:

Figs. S1 and S2
Text S1
Legends for data files S1 to S7

Other Supplementary Material for this manuscript includes the following:

(available at advances.sciencemag.org/cgi/content/full/7/12/eabe2778/DC1)

Data files S1 to S7

Supplementary Figures S1 and S2

Figure S1. Full-page images of all 13 musculoskeletal models used in this study (also in Figures 1, 2).
Right hindlimbs in lateral view. Red lines are MTUs; blue points are "via points"; bones underlie these.

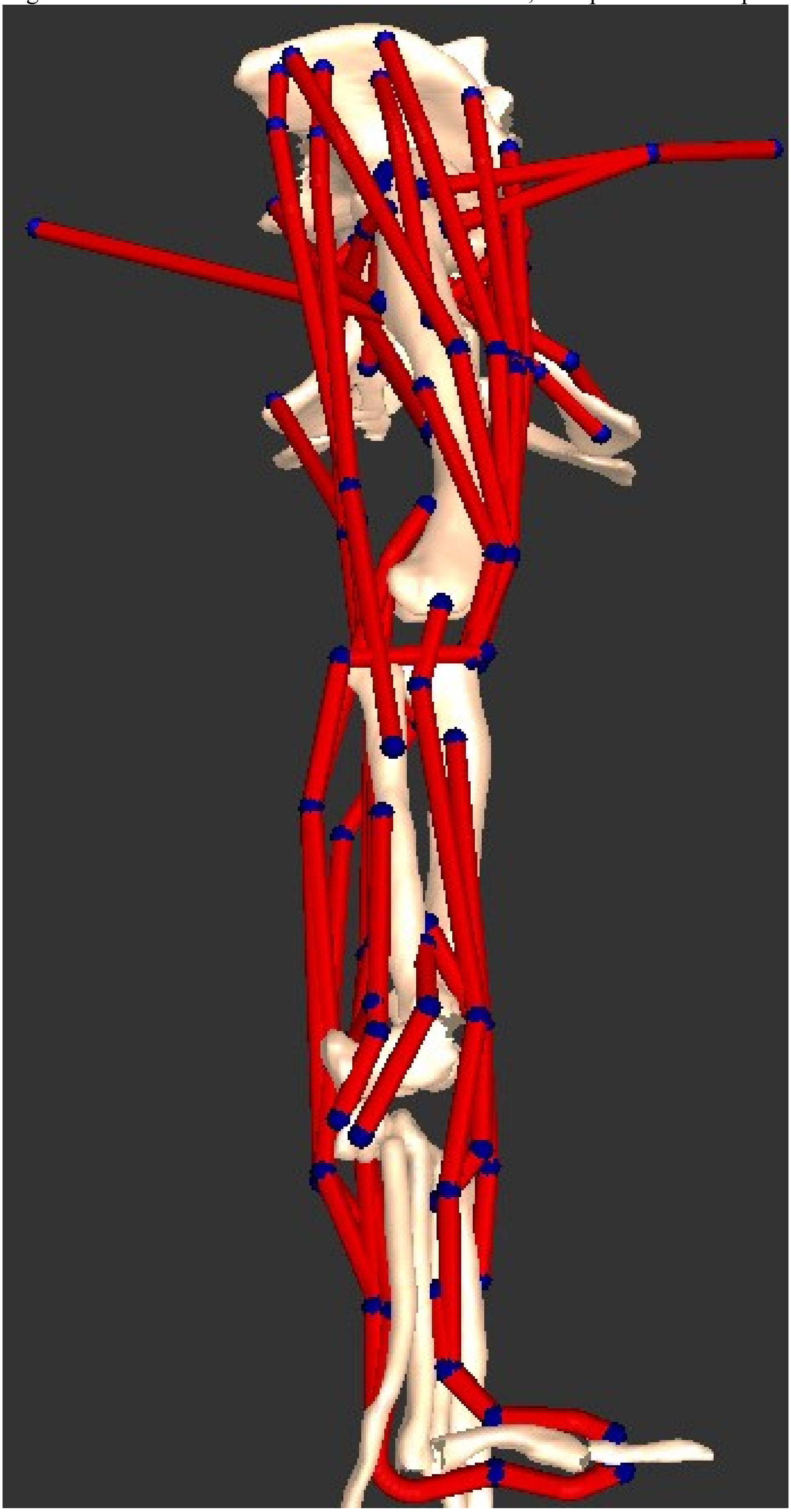

Crocodylus

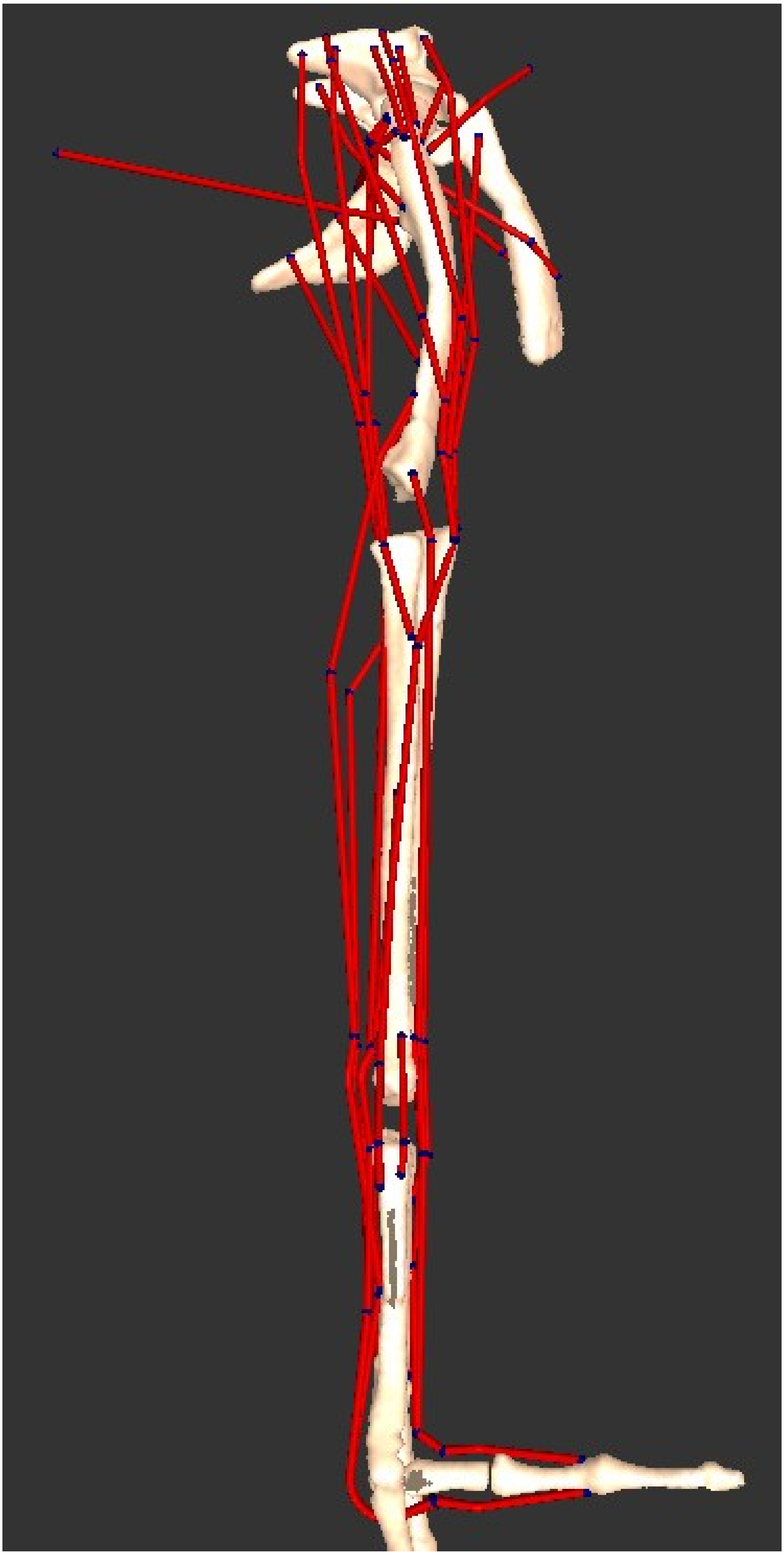

Marasuchus

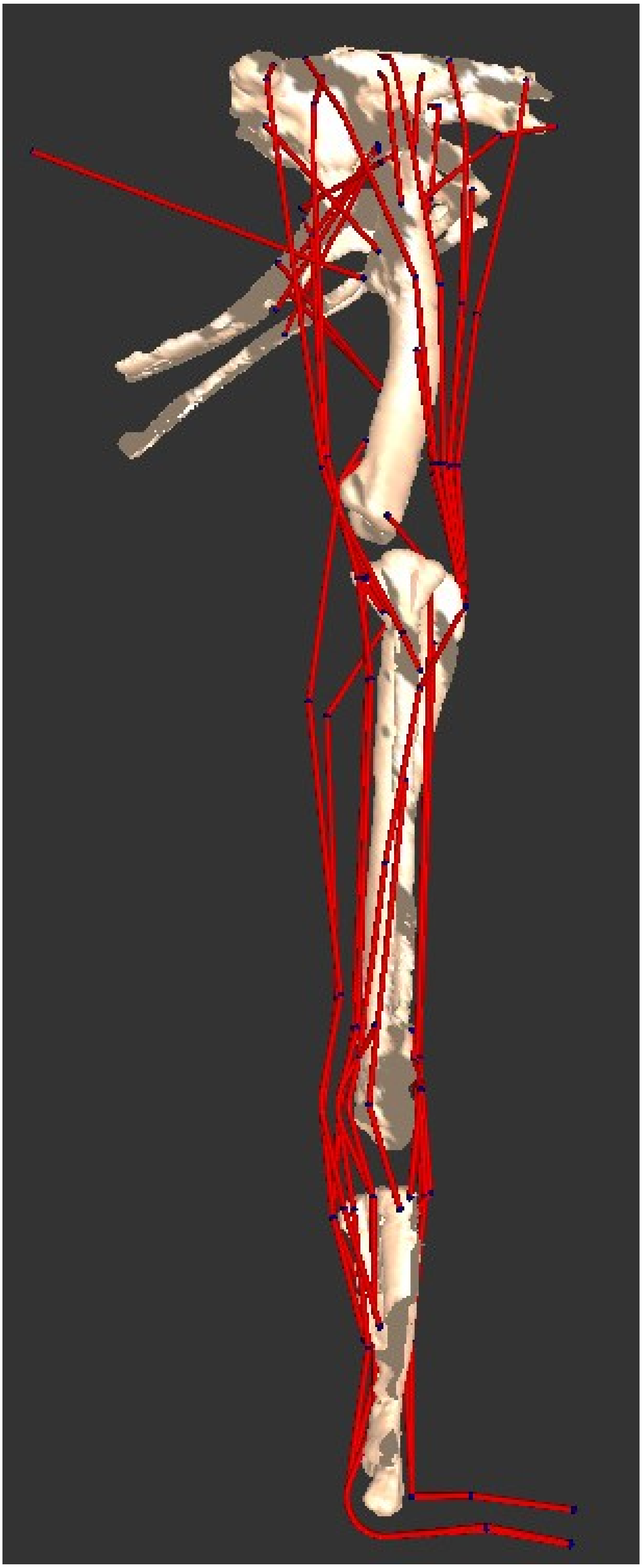

Lesothosaurus

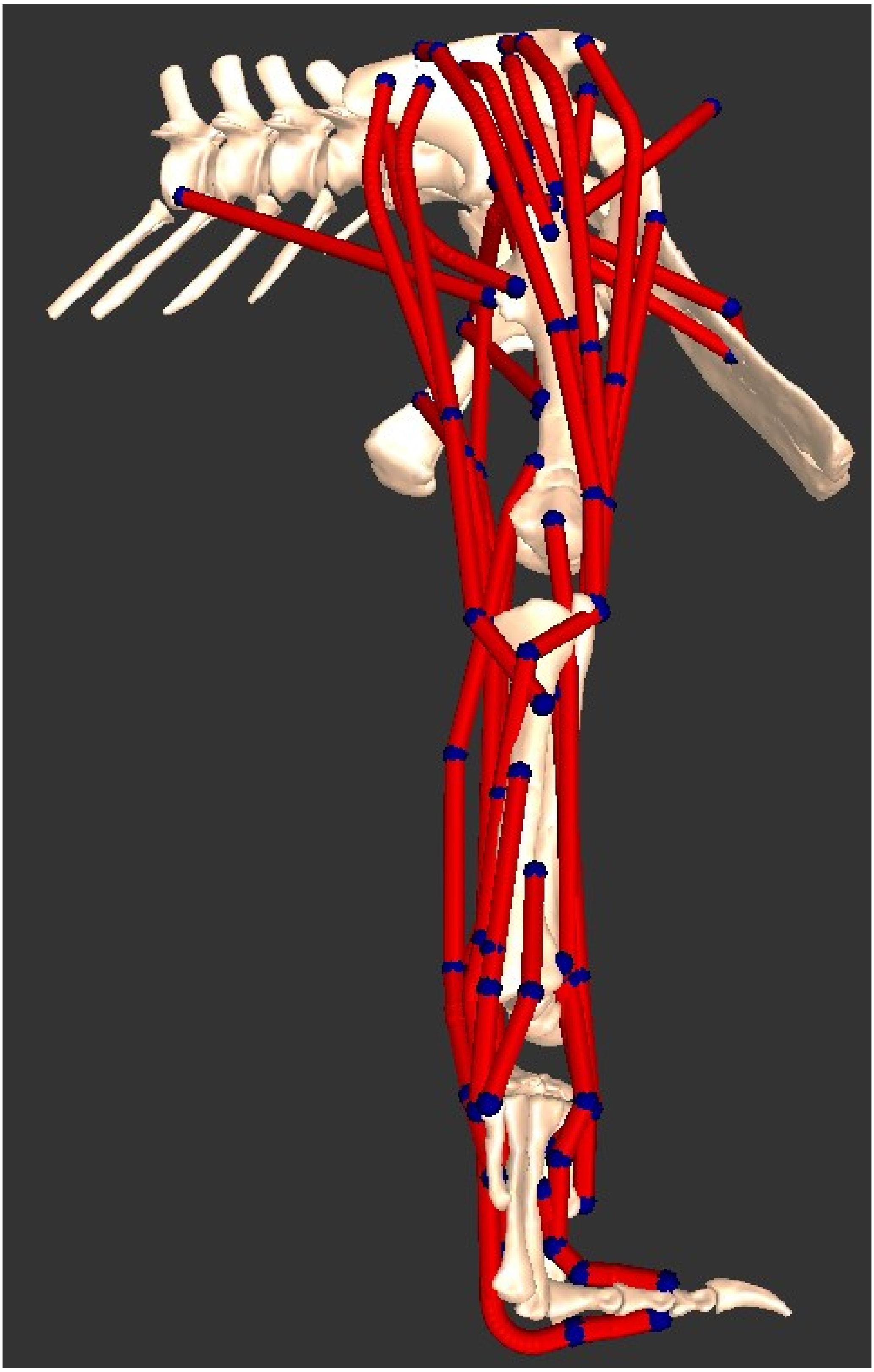

Plateosaurus

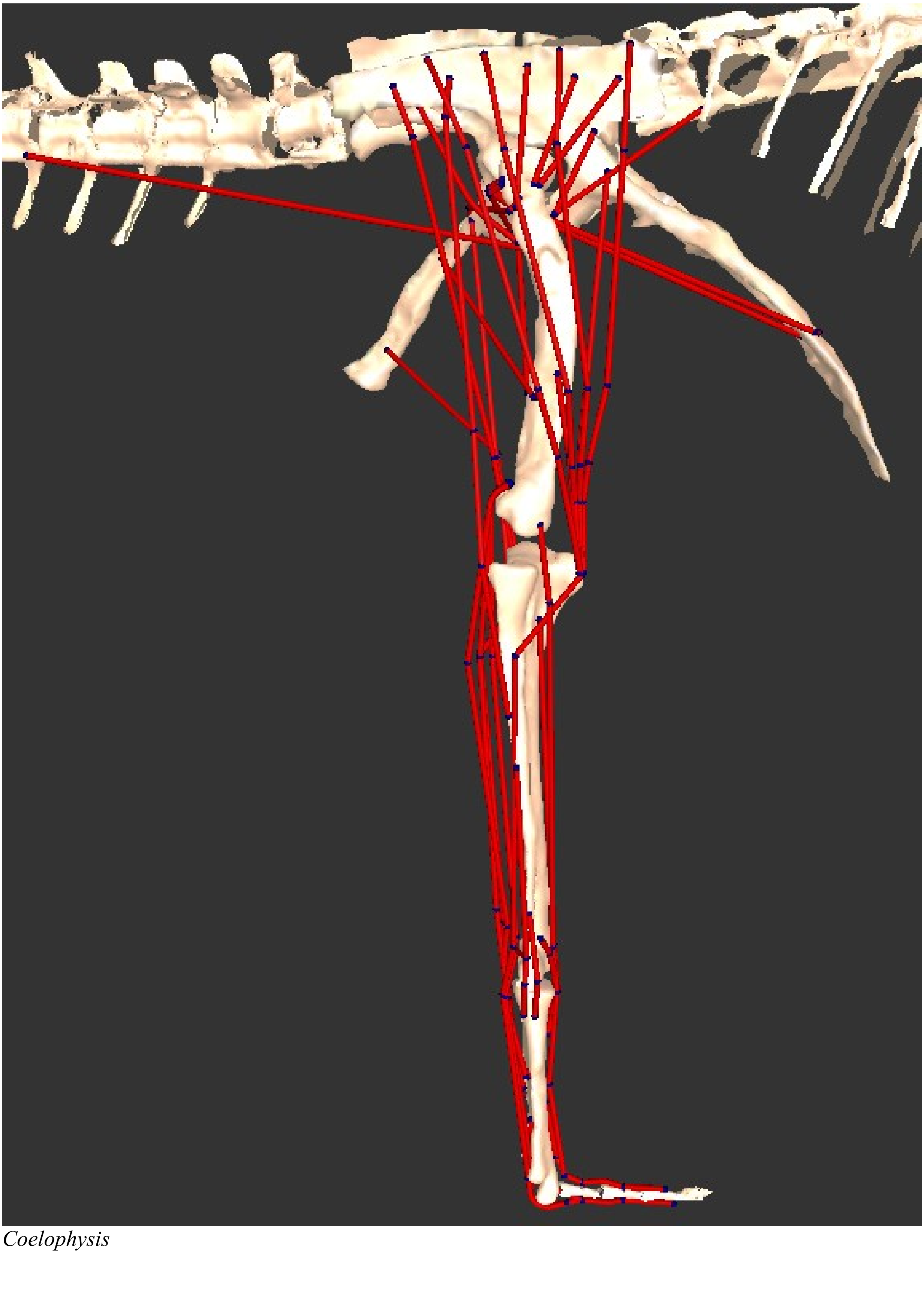

Coelophysis

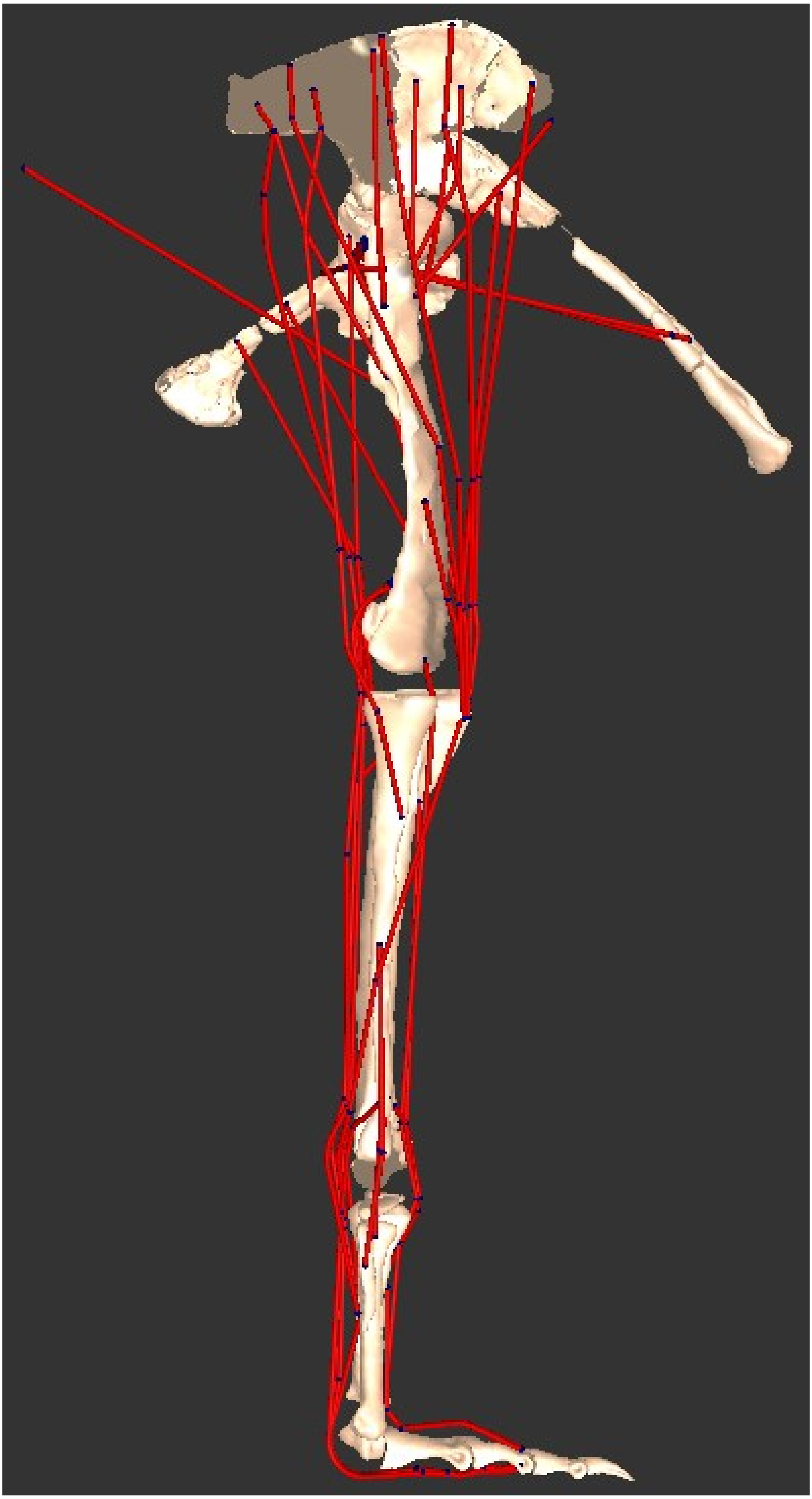

Dilophosaurus

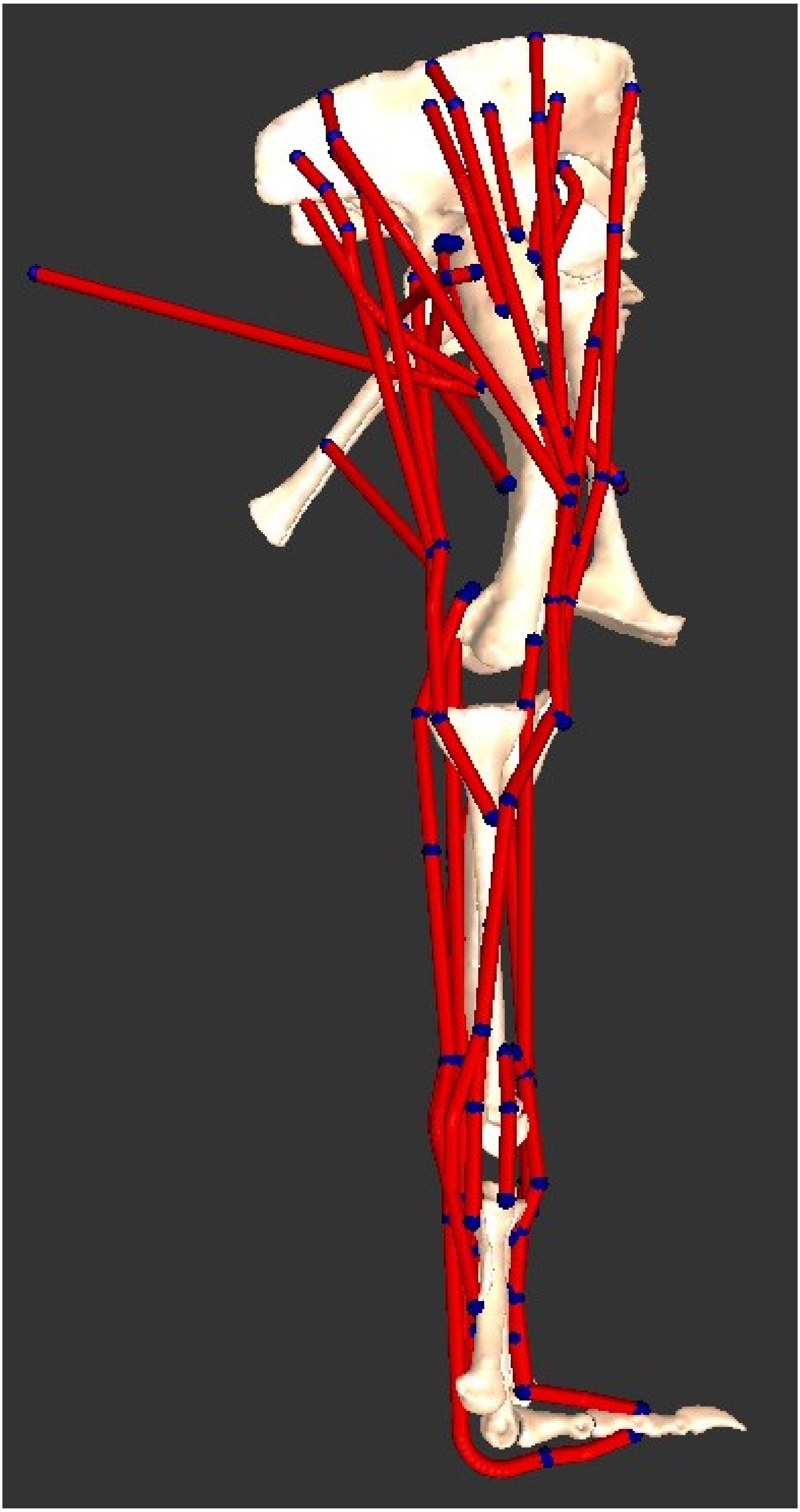

Allosaurus

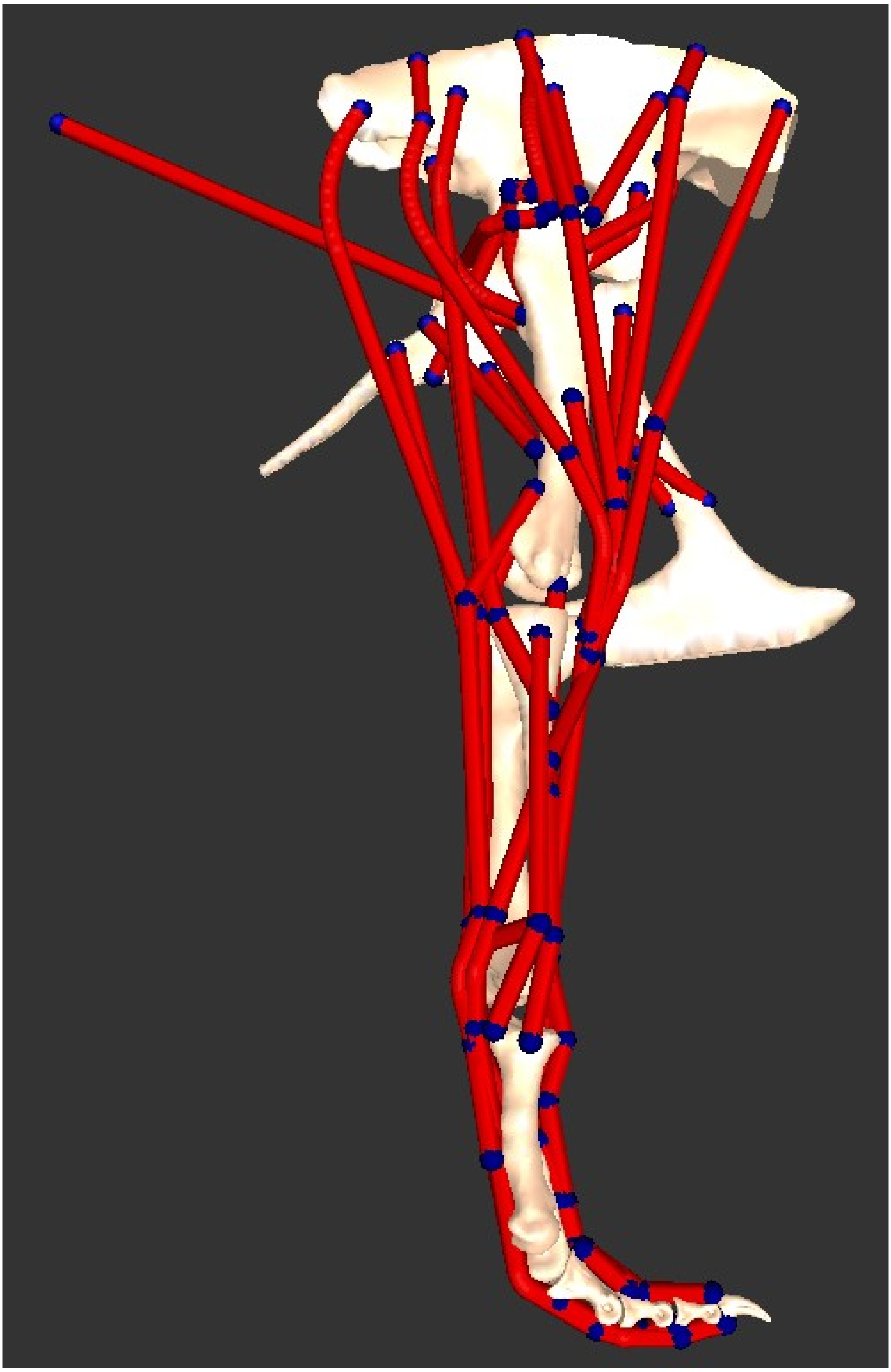

Tyrannosaurus

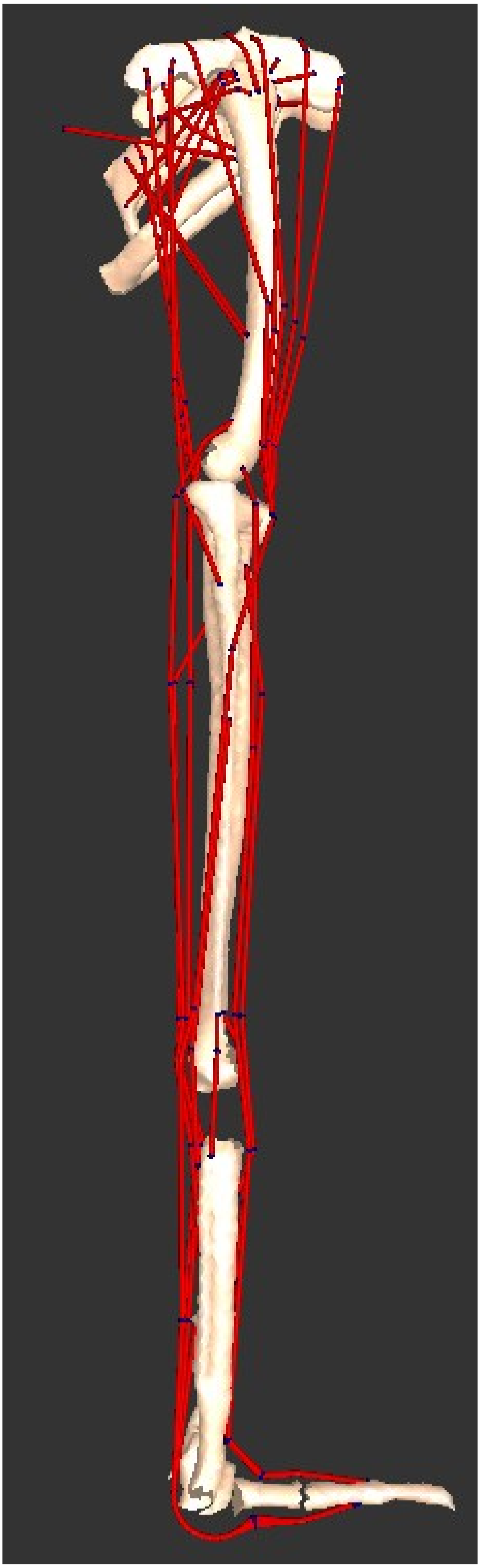

Microraptor

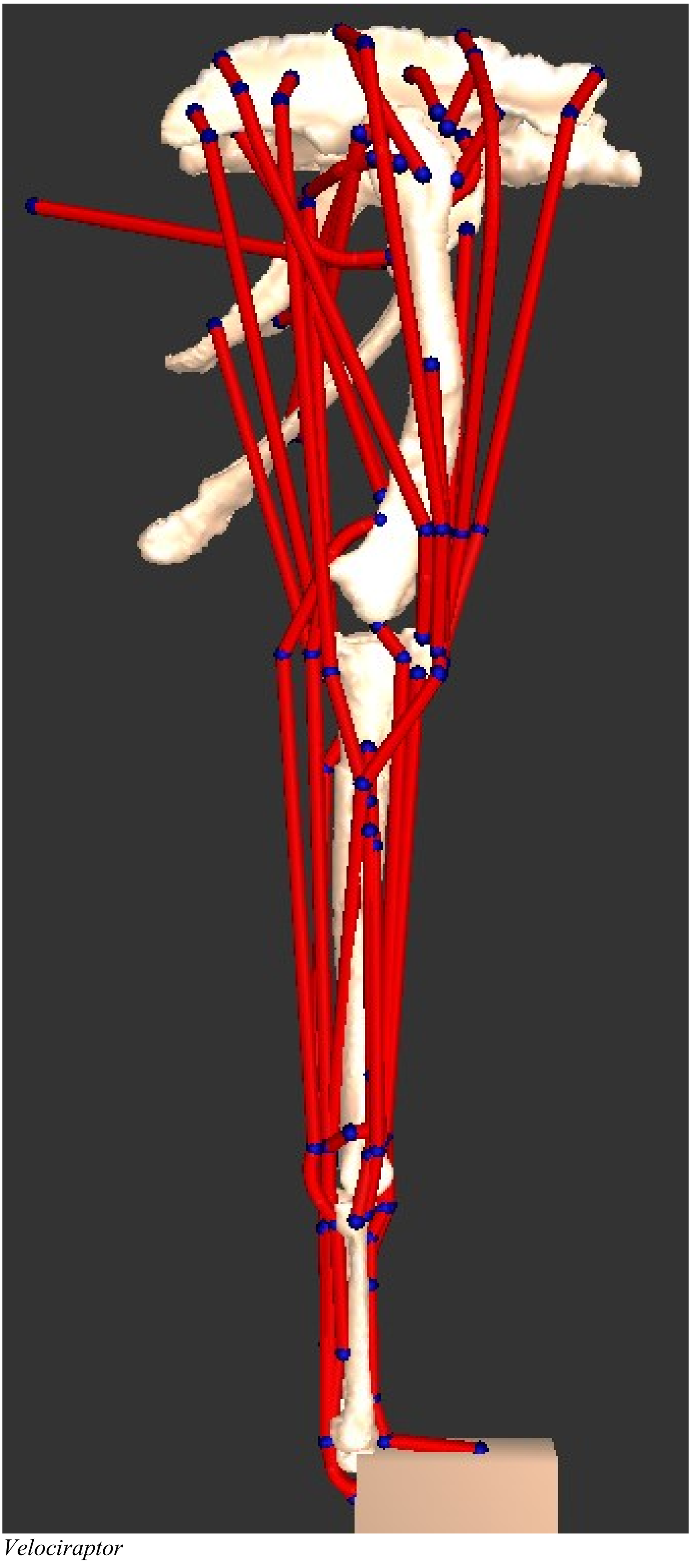
Velociraptor

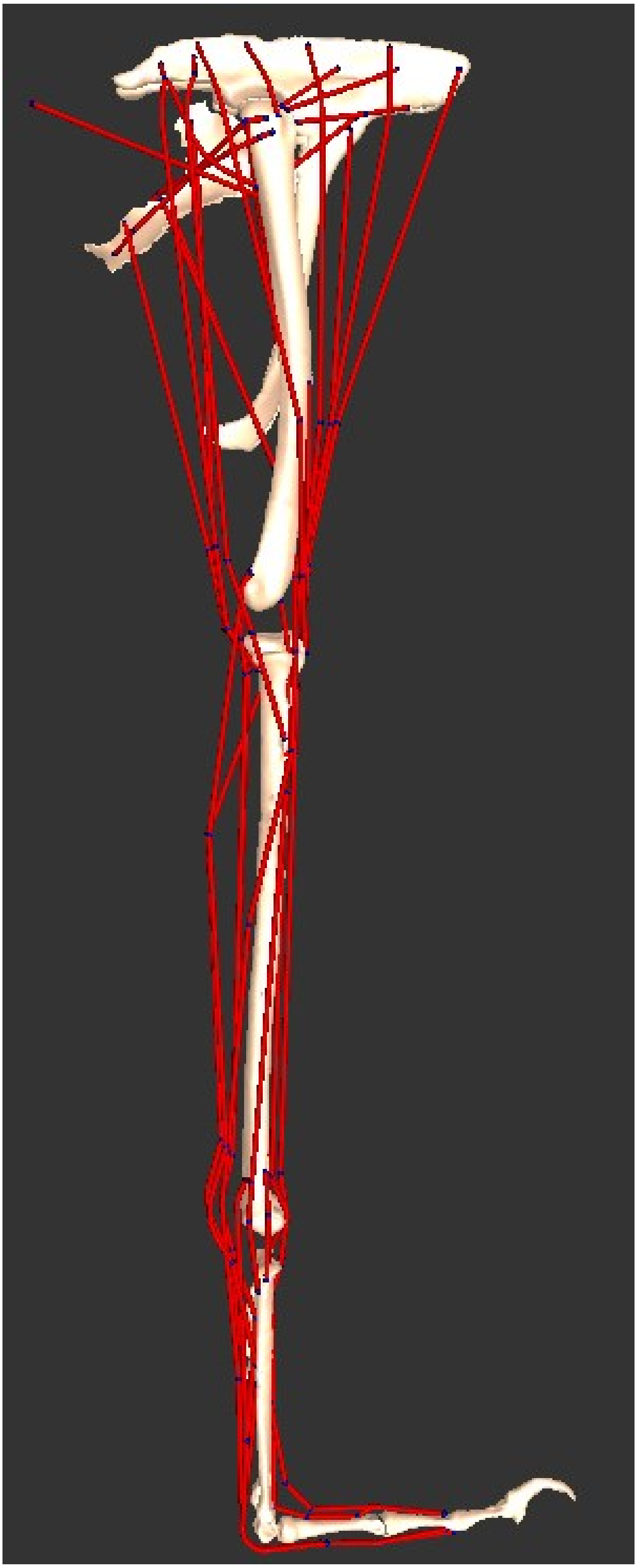

Archaeopteryx

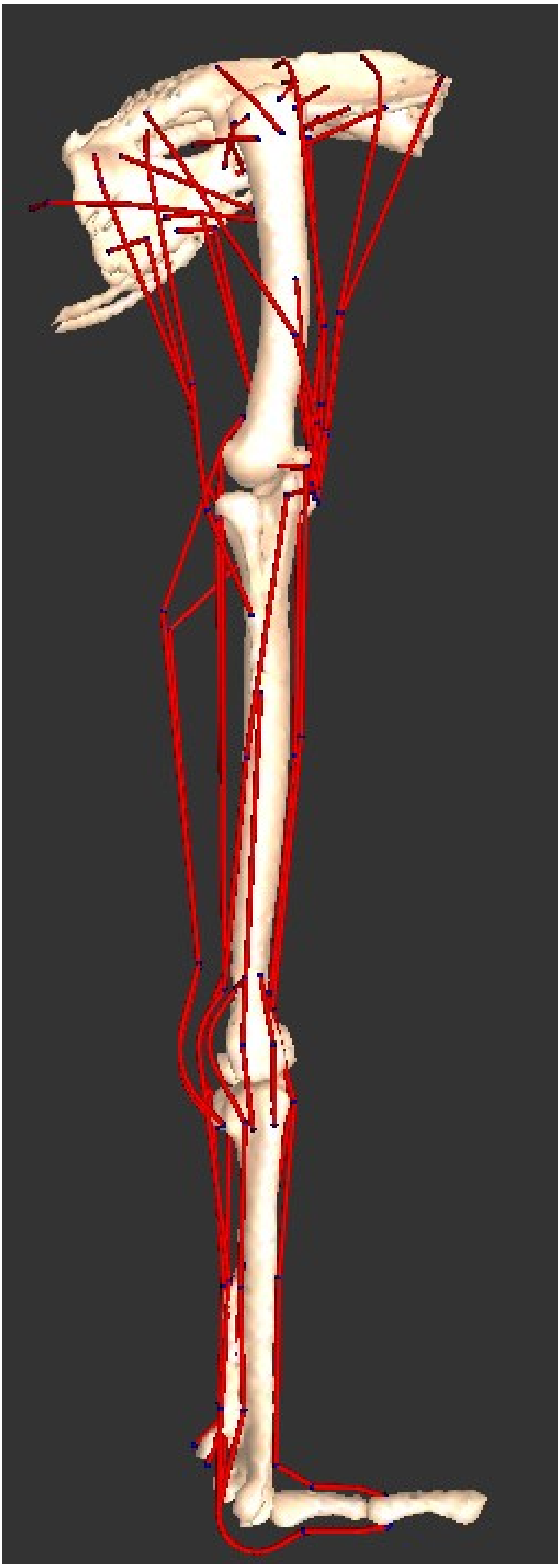

Gallus

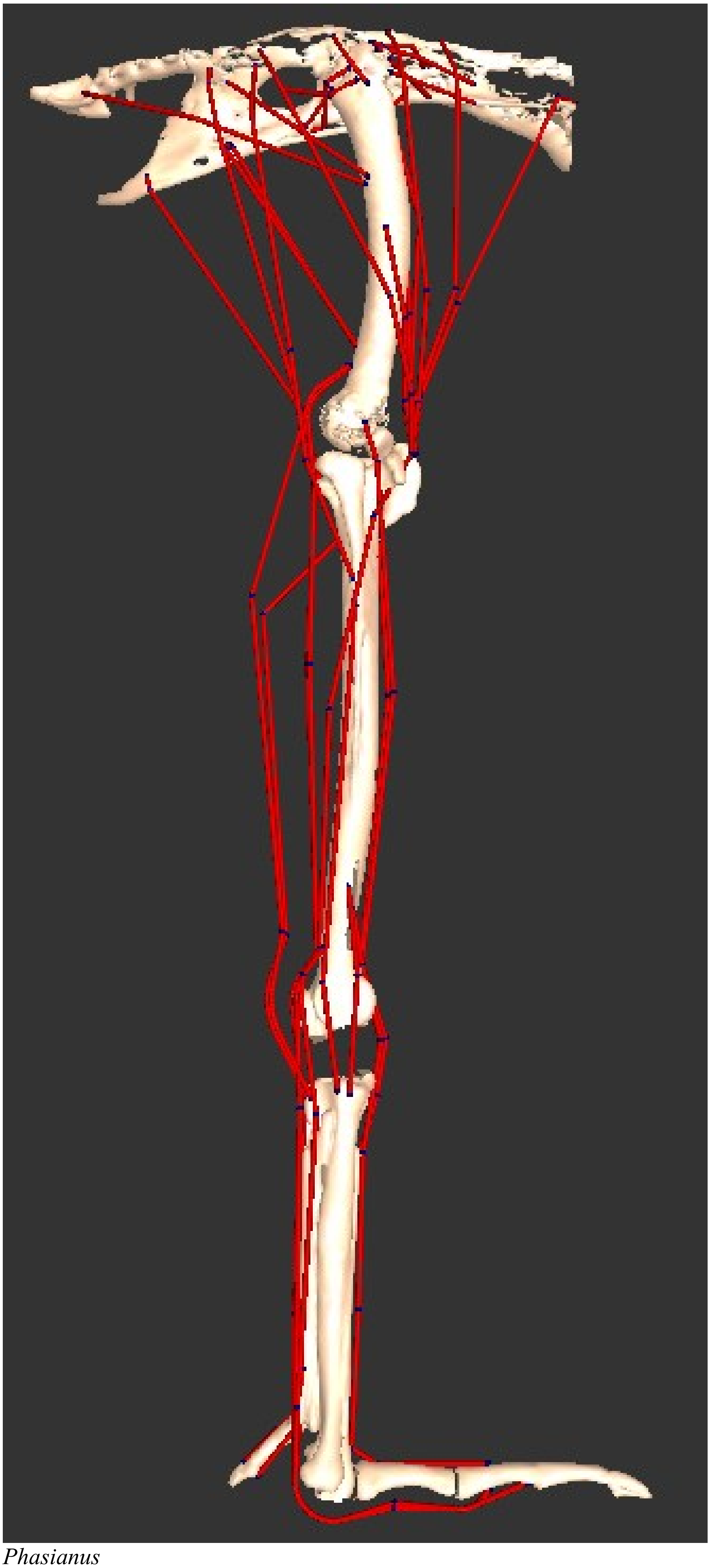

Phasianus

Figure S2. Alternative results for trends in the ratios of summed (absolute value) moment arm ACE values across the phylogeny of Archosauria (cf. Figure 8). These results are from a sensitivity analysis of branch length effects from the phylogeny (see Materials and Methods: Assumptions). (A) Changes between nodes (#1-12) of ≥20% magnitude are emphasized. Hypothesis 1 (H1) is rejected at Neotheropoda = node 5 (and increase at node 12), H2 is supported at node 5, and H3 is supported at node 5 (and nodes 8,11,12; with unexpected decreases and increases at nodes 2 and 10 respectively). + and – signs by muscle actions for H1-H3 indicate signs of (non-absolute value) moment arms. "LAR" = long-axis rotation. (B-D) Detailed changes of ratios of summed moment arms across the phylogeny of Archosauria, used for inferences in Fig. 8A. See also Figure 1. These results address H1 (B), H2 (C) and H3 (D).

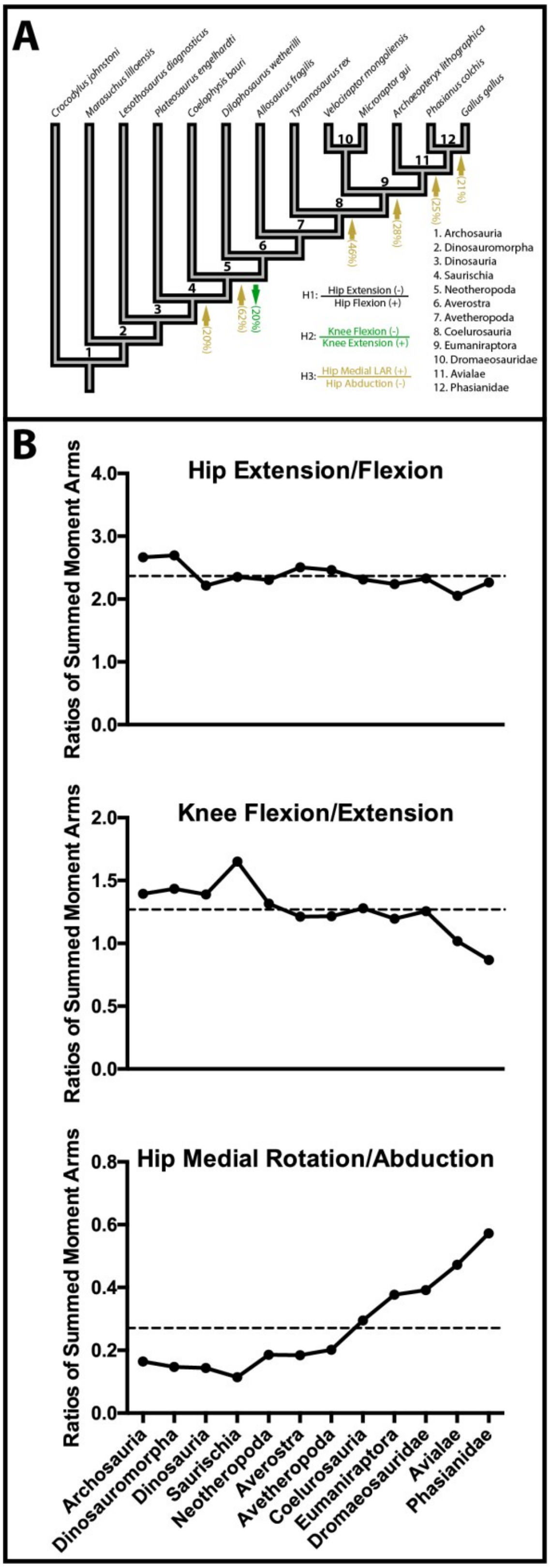

The Evolution of Pelvic Limb Muscle Moment Arms in Bird-Line Archosaurs; Allen et al.
Science Advances
Text S1. Musculo-tendon unit moment arms normalized by segment length, for ancestral nodes along the phylogeny (Figure 1), as peak and minimal values for ancestral character state estimates [ACEs].

Hip extensors (Figure 3): peaks/minima

Muscle	Peak	Minimum
CFL	-0.33	-0.16
CFB	-0.23	-0.14
ILFB	-0.24	-0.16
ADD1	-0.22	-0.17
ADD2	-0.31	-0.20
FTI1	-0.45	-0.03
FTI3	-0.36	-0.12
FTE	-0.36	-0.21
IT3	-0.15	-0.05
ISTR	-0.051	-0.011
IFE	-0.032	0.037
ITC	-0.023	0.037

Hip flexors (Figure 3): peaks/minima

Muscle	Peak	Minimum
AMB	0.17	0.070
IT1	0.42	0.12
IT2	0.22	0.0016
PIFE1	0.12	0.020
PIFE2	0.12	0.034
PIFE3	0.031	0.010
PIFI1	0.11	0.046
PIFI2	0.11	0.0041

Hip adductors (Figure 4): peaks/minima

Muscle	Peak	Minimum
ADD1	0.26	-0.013
ADD2	0.22	-0.012
FTI1	0.33	0.016
FTI3	0.064	-0.0044
ISTR	0.11	0.017
PIFE1	0.10	0.035
PIFE2	0.10	0.043
PIFE3	0.13	0.0069

Hip abductors (Figure 4): peaks/minima

Muscle	Peak	Minimum
CFB	-0.095	-0.022
ILFB	-0.14	-0.087
FTE	-0.10	-0.051
AMB	-0.020	0.035
IT1	-0.12	0.061
IT2	-0.20	-0.070
IT3	-0.19	-0.12
PIFI1	-0.070	0.038
PIFI2	-0.035	0.013
IFE	-0.15	-0.094
ITC	-0.12	-0.031

Hip lateral rotators (Figure 5): peaks/minima

Muscle	Peak	Minimum
CFL	-0.064	-0.013
CFB	-0.070	-0.026
ILFB	-0.017	-0.00038
ADD2	-0.016	0.00087
FTI1	-0.018	0.014
ISTR	-0.15	-0.095
AMB	-0.011	0.031
IT1	-0.030	0.031
IT2	-0.040	0.0058
IT3	0.070	-0.26
PIFE1	-0.072	-0.00086
PIFE2	-0.066	0.018
PIFE3	-0.13	-0.012
IFE	-0.032	~0

Hip medial rotators (Figure 5): peaks/minima

Muscle	Peak	Minimum
ADD1	0.012	-0.012
FTI3	0.030	-0.0015
FTE	0.048	0.0061
PIFI1	0.052	0.025
PIFI2	0.14	0.068
ITC	0.11	-0.0078

Knee extensors (Figure 6): peaks/minima

Muscle	Peak	Minimum
AMB	0.091	0.049
IT1	0.12	0.071
IT2	0.11	0.064
IT3	0.090	0.056
FMTI	0.090	0.052
FMTE	0.088	0.058
EDL	0.061	0.0043
TA*	0.023	~0

*EDL in Phasianidae

Knee flexors (Figure 6): peaks/minima

Muscle	Peak	Minimum
ILFB	-0.12	-0.067
FTI1	-0.12	-0.0089
FTI3	-0.12	-0.075
FTE	-0.12	-0.071
GL	-0.087	-0.030
FDL	-0.085	-0.032
FHL	-0.082	-0.035

Ankle plantarflexors (extensors) (Figure 7): peaks/minima

Muscle	Peak	Minimum
AMB*	-0.14	-0.091
GL	-0.19	-0.13
GM	-0.18	-0.12
FDL	-0.14	-0.090
FHL	-0.13	-0.093
FL	-0.042	-0.0035
FL **	-0.10	-0.0066

*distal tendon; **secondary tendon

Ankle dorsiflexors (Figure 7): peaks/minima

Muscle	Peak	Minimum
EDL	0.12	0.070
EHL	0.11	0.012
TA	0.12	0.084
FB	0.058	-0.015

Ratios of Summed Moment Arms (Figure 8): peaks/minima

Muscle	Peak	Minimum
Hip extensor/flexor	3.27	1.68
Knee flexor/extensor	1.59	0.82
Hip medial LAR/abduction	0.33	0.073

Supplementary Data Files

Data file S1. Information on specimens used for musculoskeletal models. Includes museum details, general assessment of specimen (hindlimb) completeness, scanning and reconstruction methods used, body mass (from *12*), and hindlimb dimensions used for moment arm normalization.

Data file S2. Study taxa branch lengths and node divergence times used in this study (also see Figure 1).

Data file S3. Detailed results from phylogenetic analysis of hip extensor and flexor mean MTU moment arm data. Worksheets show data (values per node from ACE analysis, per muscle) for extensors, flexors and then the ratio of their summed values per node, with changes of ≥10% and ≥20% (as per Figure 8) in red font or highlighted. Highlighted "change" cells summarize total change from Archosauria to Phasianidae nodes.

Data file S4. Detailed results from phylogenetic analysis of knee flexor and extensor mean MTU moment arm data. See caption of Data file S3 for further details.

Data file S5. Detailed results from phylogenetic analysis of hip long-axis rotator (LAR; medial and lateral) and abduction/adduction (ABAD) mean MTU moment arm data. See caption of Data file S3 for further details.

Data file S6. Detailed results from phylogenetic analysis of ankle (dorsi)flexor and extensor (plantarflexor) mean MTU moment arm data. See caption for Data file S3 of further details.

Data file S7. Scripts and associated files used for analyses in OCTAVE and R (.zip archive). README text file explains organization.
