# Supplementary figures and images for: The evolution of pelvic limb muscle moment arms in bird-line archosaurs
Source: Sci Adv. 2021 Mar 19;7(12):eabe2778. doi: 10.1126/sciadv.abe2778 (PMC7978429; doi:10.1126/sciadv.abe2778)

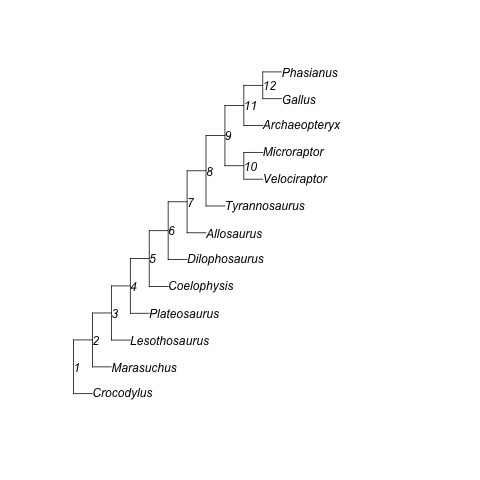

Supplement: Data file S7 [file abe2778_Data_file_S7.zip › supp data/data_processing_files/dino_moment_arms_Sun_Jun_07_14-26-54_2020-MAC_2_tree.jpeg]
